# Functional Genomic Evidence for Candidate Small Viral RNA-Mediated Epigenetic Interference in SARS-CoV-1 and SARS-CoV-2

**DOI:** 10.34133/csbj.0148

**Published:** 2026-07-02

**Authors:** Amber R. Paulson, Vincent Montoya, Jeffrey B. Joy

**Affiliations:** ^1^ BC Centre for Excellence in HIV/AIDS, Vancouver, BC, Canada.; ^2^ Department of Medicine, University of British Columbia, Vancouver, BC, Canada.; ^3^ Bioinformatics Programme, University of British Columbia, Vancouver, BC, Canada.

## Abstract

Small viral RNAs (svRNAs) have emerged as key factors in host adaptation and virulence, with evidence of their presence in both SARS-CoV-2 and SARS-CoV-1. Here, using functional genomics, we identify 6 candidate svRNA-triplex-forming oligonucleotides (svRNA-TFOs) in SARS-CoV-2 and, through comparative analyses with SARS-CoV-1, delineate evolutionary pathways for svRNA-TFOs in Sarbecoviruses. In addition to the significant enrichment of candidate svRNA-TFO enhancer-gene targets among differentially expressed genes during SARS-CoV-2 infection, these svRNA-TFOs also show nonrandom associations with over 25% of the recombination breakpoints and hotspots in the Wuhan-Hu-1 genome. Small RNA-sequencing reveals highly abundant svRNA-TFO-N derived from the nucleocapsid of SARS-CoV-2. Using synonymous-site conservation analysis, we show that svRNA-TFO-N and other svRNA-TFOs are conserved among both SARS-CoV-2- and SARS-CoV-1-related lineages. Furthermore, BLASTn reveals that svRNA-TFO-N and svRNA-TFO-S.1 share sequence homology against various mammalian genomes, which supports a proposed mechanism of host adaptation. Finally, based on RNA structural modeling, we identify variant of concern-associated S:D614G and N:D377Y mutations, which may alter precursor structures predicted for svRNA-TFO-S.1 and svRNA-TFO-N, respectively. Collectively, these findings provide support for the hypothesis that svRNA-mediated epigenetic interference contributes to virulence in SARS-CoV-2 and SARS-CoV-1.

## Graphical Abstract

**Figure:**
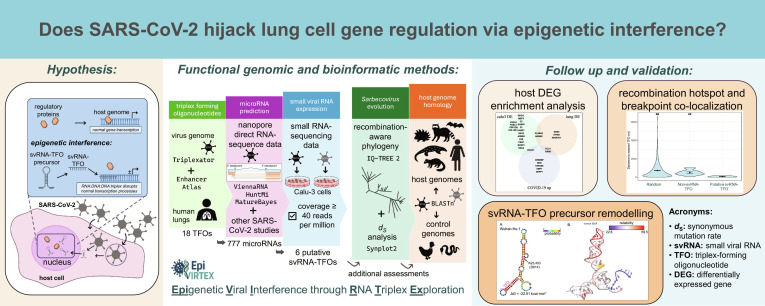

## Introduction

Small viral RNAs (svRNAs) are emerging as major players in the pathogenesis of viral infections [1–4], including in SARS-CoV-2, which produces both microRNAs (miRNAs) and nuclear-acting miRNAs (namiRNAs) that regulate host gene expression [5–9]. More broadly, small noncoding RNAs (ncRNAs) less than 200 nucleotides (nts) in length are well-established regulators of gene expression [10–12], with canonical ~22-nt miRNAs being the most comprehensively studied; these single-stranded RNAs typically act through the RNA-induced Silencing Complex to target the 3′ untranslated regions (UTRs) of messenger RNAs [13–16]. During SARS-CoV-2 infection, several virus-derived miRNAs, including those from the ORF7a and nsp2 regions, have been shown to modulate host immune response [5,8] and correlate with disease severity, respectively [9]. In another study, an ORF8-encoded miRNA was shown to be abundant on nasal swabs collected from COVID-19 patients and suggested to act as a decoy by interacting with host miRNAs [6]. Additionally, SARS-CoV-2 also produces 5 human-identical sequence (HIS) namiRNAs that act through nuclear Argonaute 2 to target human enhancers and up-regulate adjacent or distal genes, including cytokine genes and *hyaluronan synthase 2* [7]. While Sarbecoviruses such as SARS-CoV-1 and SARS-CoV-2 have been intensively studied, the evolutionary processes underlying their emergence into the human population remains unresolved, underscoring the importance of understanding the potential for svRNA-mediated host interactions in this group.

While namiRNAs are generally known to target enhancers [17–19], increasing attention is now being directed toward the role of RNA triplex-forming oligonucleotides (TFOs), which can also target enhancers and other important regulatory regions in the genome [20–22]. Endogenous TFOs in humans can be encoded on long or small ncRNAs and range from approximately 12 to 30 nts in length [23–32]. Their sequence-specific binding of polypurine DNA within the major groove is a result of 3 TFO motif types (GA, GU, or CU), which form RNA:DNA:DNA triple helices (triplexes) via Hoogsteen base-pairing forces [33–36]. Emerging *in silico*, biophysical, and *in vitro* evidence reveals enrichment of TFO-binding sites within promoter regions, 5′ UTRs, super-enhancers, and repetitive elements [20,22]. One study showed that these sites are preferentially positioned within nucleosome-depleted regions and active regulatory elements where chromatin architecture promotes triplex formation [21]. These findings support a regulatory role for RNA:DNA:DNA triplexes at key genomic loci. Despite the growing recognition of TFOs in eukaryotic gene regulation [37–39], their role in host–virus interactions remain largely unexplored. Host miRNAs, for instance, can form triplexes with integrated provirus genomes to regulate lentivirus latency and persistence [40] and inhibit human immunodeficiency virus 1 replication [41], and influenza A virus can trigger endogenous triplex formation that represses transcription of the gene for beta interferon [42]. Nevertheless, the potential contribution of exogenous virus-derived TFOs as a novel class of svRNA has not been investigated, making this study the first to explore this possibility.

Production of structural and accessory proteins in coronaviruses involves a unique mechanism of discontinuous transcription and results in a nested set of subgenomic RNAs (sgRNAs) [43,44]. A transcriptome-wide study combining DNA Nanoball sequencing and nanopore-based direct RNA-sequencing (DRS) revealed that SARS-CoV-2, beyond production of canonical sgRNAs, also generates a variety of noncanonical sgRNAs with putative RNA modifications at an AAGAA-like motif [43]. Additionally, 3 miRNA-like small RNAs are produced by SARS-CoV-2, as previously mentioned [5,6,8]. Although less studied, the SARS-CoV-1 transcriptome also encodes 3 miRNA-like small RNAs derived from nsp3 and N-ORF regions validated by Morales *et al.* [4], one of which modulates lung pathology in mice. Together, these findings highlight that *Sarbecovirus* transcriptomes encode diverse RNA products with possible regulatory functions, yet it remains unclear whether such factors drive evolution or contribute to cross-species transmissibility among this high-risk group. To address this gap, we leverage genomic resources from diverse Sarbecoviruses alongside comparative small RNA-sequencing (RNA-seq) of SARS-CoV-2 and SARS-CoV-1 during Calu-3 cell infection [45].

We hypothesized that SARS-CoV-2 can produce svRNAs that are capable of triplex-mediated epigenetic interference, thereby altering the expression of distal host genes relevant to infection. Epigenetic dysregulation is already suspected to be central to COVID-19, where distinct genome-wide DNA methylation profiles are detected in the blood of patients with severe COVID-19 [46,47]. Also, restructuring of the chromatin architecture by SARS-CoV-2 has been shown [48] and the virus produces nuclear-acting proteins such as ORF8 that targets the host nucleosome by mimicking histone and disrupts epigenetic regulation [49]. Despite these clues, and the presence of viral namiRNAs in the nucleus [7], the role of triplex-forming svRNAs in SARS-CoV-2 has not been investigated, which motivates this study.

Importantly, previous studies validating miRNA-like small RNAs in SARS-CoV-2 and SARS-CoV-1 [4,5,8,9] quantified their relative abundance using small RNA-seq library preparation methods that optimize for capture of canonical miRNAs with intact 5′ phosphate and 3′ hydroxyl groups [50–52]. However, because the RNase L pathway plays a central role during SARS-CoV-2 infection [53–56], we focused on whether svRNAs might instead arise from RNase-mediated degradation products. Using small RNA-seq data from an adapter ligation-free library preparation method [45] that enables better recovery of RNase-derived fragments [50] carrying 5′ hydroxyl groups and 2′ or 3′ phosphates [51,52,57], we identified 6 candidate svRNA-TFOs of interest and investigated their conservation and evolution across SARS-CoV-2, SARS-CoV-1, and other Sarbecoviruses. We further assessed for enrichment of their enhancer-gene targets among differentially expressed genes in SARS-CoV-2-infected human bronchial epithelial cells and a lung-derived cell line, and from the whole blood from severe COVID-19 patients. We predicted that 2 svRNA-TFO precursor structures show mutation-associated changes in variants of concern (VoCs) and that all 6 candidate svRNA-TFOs exhibited nonrandom associations with known genomic recombination breakpoints and hotspots in the Wuhan-Hu-1 genome. To support this analysis, a functional genomics framework, “Epigenetic Viral Interference through RNA Triplex Exploration” (Epi-VIRTEX), was developed that combines machine learning for viral miRNA prediction, nanopore DRS data for observable SARS-CoV-2 sgRNAs [43], small RNA-seq [45], and recombinant-aware phylogenetics across *Sarbecovirus* genomes isolated from humans, pangolins, and greater horseshoe bats.

## Materials and Methods

### Epi-VIRTEX for functional genomics of SARS-CoV-2

Epi-VIRTEX is a functional genomics pipeline that integrates virus-related RNA-seq datasets with sequence-based RNA structure, and miRNA and triplex predictions. To identify candidate svRNA-TFOs requiring further investigation from SARS-CoV-2, small RNA-seq data collected during SARS-CoV-2 and SARS-CoV-1 infection of Calu-3 cells were re-analyzed [45]. Additional miRNA predictions were generated using nanopore DRS data of SARS-CoV-2 for the observable sgRNAs collected from Vero cells at 24 hpi [43]. Three human transcriptome studies measuring differential gene expression in response to SARS-CoV-2 infection from Calu-3 cells [45,58], undifferentiated human bronchial epithelial cells [58], and the whole blood of COVID-19 patients [59] formed the basis for an enrichment-based validation.

### TFO and microRNA prediction in SARS-CoV-2

To investigate epigenetic interference as a potential virulence mechanism in SARS-CoV-2, putative TFOs from the Wuhan-Hu-1 reference genome were identified from the human lung-associated enhancer sequences from EnhancerAtlas 1.0 [60] using Triplexator [61]. The Triplexator algorithm implements sequence matching based on canonical triplex-formation rules and was implemented using the lowest allowable size limit of 12 nt.

Next, sequences representing 477 observed SARS-CoV-2 sgRNAs during infection of Vero Cells [43] were obtained from the University of California, Santa Cruz SARS-CoV-2 Genome Browser [62,63]. After collapsing redundant sequences at a 99% identity threshold using CD-HIT (v4.8.1-2019-0228), 153 nonredundant sgRNA sequences were carried forward for further analysis [64]. Using SeqKit (v0.12.1) [65], these sequences were fragmented with a window-size and sliding-step length of 120 and 20 nt, respectively. Each fragmented sequence was then analyzed for its structural thermal stability with the ViennaRNA (v2.4.14) RNAfold algorithm [66] to identify 5,670 structures meeting the −20 kcal mol^−1^ cutoff threshold for optimum predicted minimal free energy (MFE). From these RNA structures, 223 pre-miRNA hairpins were predicted by the machine learning tool HuntMi trained on a virus-specific geometric mean-optimized training set [67]. Lastly, to predict mature miRNAs, a naïve Bayes classifier MatureBayes was used to identify 446 mature miRNAs from SARS-CoV-2 [68]. Nonredundant representatives of these miRNAs, along with 331 other previously predicted SARS-CoV-2 miRNAs from genome-based studies [69–73] were mapped to the Wuhan-Hu-1 reference genome to identify candidate svRNA-TFOs, along with sufficient small RNA-seq coverage (see below). A number of miRNAs or namiRNAs functionally characterized in SARS-CoV-2 [5–8] and SARS-CoV-1 [4] were also mapped against the respective reference genomes (Wuhan-Hu-1 or Tor2) and used for comparative purposes with small RNA-seq.

### Small RNA-seq coverage and normalization

To identify potential svRNA-TFOs being produced within 24 hpi, a small RNA-seq cell-based experiment using Calu-3 lung cancer epithelial cells was combined with the miRNA predictions from this, and other studies, for the SARS-CoV-2 Wuhan-Hu-1 genome described above. The small RNA-seq data collected at 4, 12, and 24 hpi were downloaded from the National Center for Biotechnology Information (NCBI) GeoBank (GSE148729) on 2021 November 15 (SARS-CoV-2) and 2024 January 21 (SARS-CoV), along with the corresponding mock-infected and uninfected controls [45]. Following a similar 2-pass trimming approach described in the originating paper but instead using Flexbar (v3.0.3) [74], the first 3 nt on the 5′ end were removed, the 3′ end was trimmed to remove bases with a quality score of <30, and the Illumina small TruSeq adapter was removed from the 3′ end. On the second-pass, the polyA adapter was removed if there was an overlap of 10 nt with no mismatches. Trimmed/filtered sequences were next aligned to the SARS-CoV-2 reference genome (NC_045512.2; Wuhan-Hu-1) using Bowtie 2 (v2.3.4.1) [75].

Alignment files were sorted using Samtools (v1.10) [76] and reads per million (RPM) coverage was determined with bedtools (v2.27.1) [77]. For visualization purposes, ggplot2 (v3.3.3) [78] was used to show coverage of 2 biologically replicated libraries over the presumed svRNA-TFO precursor RNA structure regions (see methods below), or else the coverage plots were centered on the TFO. To identify candidate svRNA-TFOs, regions encoding predicted TFOs were assessed for small RNA-seq coverage. To mitigate for a high false positive rate stemming from the large number of mature miRNA predictions from the SARS-CoV-2 sgRNAs (see above), candidate svRNA-TFOs were required to reach at least 40 RPM coverage within 25 nt of predicted TFOs in both replicates at 12 or 24 hpi. This is considered an appropriate operational threshold for candidate svRNA prioritization because prior small RNA-seq studies used a range of RPM thresholds from 10 to 100 to predict endogenous miRNAs [79–81], which would be expected to occur in relatively higher abundance. To compare RPM small RNA-seq coverage between SARS-CoV-1 and SARS-CoV-2, the 12 hpi time point was selected as the 2 viruses produce different viral particle counts between at 12 hpi and coverage is lower at 4 hpi [45].

### Phylogenetic reconstruction, synonymous-site conservation analysis, motif searches, and recombination breakpoint/hotspot analysis

Using the DECIPHER (v2.26.0) [82], amino acid-based multiple sequence alignments (MSAs) were made from 76, 85, 80, 54, and 76 nonredundant translated nucleotide sequences derived from the nsp2, nsp3, S-, E-, and N-ORF regions, respectively. The sequences used in this analysis included SARS-CoV-2 (Wuhan-Hu-1) and SARS-CoV-1 (Tor2) reference genomes, the genomes of 96 other bat- and pangolin-infecting *Sarbecovirus* available from the NCBI GenBank and the Global Initiative on Sharing All Influenza Data repositories (Table S1), and the SARS-CoV-2 most-recent reconstructed ancestor (MRCA) genome sequence [83]. Sequences containing ambiguous bases were not included in the alignments.

To improve alignment of the nsp3 ORF region, the MRCA nsp3-associated sequence was aligned secondarily using Clustal Omega (v1.2.2) with default settings in Geneious Prime (v2023.2.1). To achieve the S-ORF alignment, sequences derived from the following accessions were omitted: KF294457.1, DQ412043.1, DQ648857.1, KJ473815.1, and NC_014470.1. Following MSA, the nsp2, nsp3, and S-ORF regions were partitioned according to the location of previously identified recombination breakpoints in Wuhuan-Hu-1 [84]. Next, using IQ-TREE 2 (v1.61) [85], phylogenetic analysis was performed on the aligned E- and N-ORF and partitioned nsp2, nsp3, and S-ORF sequences by best-fit substitution model selection according to the Bayesian information criterion. Node confidence was tested using 10,000 ultrafast bootstraps for each of the phylogenies generated.

Next the tip orders were extracted from each of the phylogenies to implement Synplot2 (v2014) analysis for the nsp2, nsp3, S-, E-, and N-ORF regions, along with the amino acid-based transcribed alignments. Synplot2 is an algorithm designed to analyze protein-coding regions from RNA viruses to identify statistically significant reductions in the variability at synonymous sites as an indicator for overlapping functional noncoding elements [86]. Also, segments of the alignments containing TFOs from the 6 candidate svRNA-TFOs were analyzed using the Multiple Em for Motif Elicitation (MEME) Suite (v5.5.9) [87]. To identify conserved motifs with MEME, all *Sarbecovirus*-related sequences were analyzed either together (svRNA-TFO-N, svRNA-TFO-S.1, svRNA-TFO-S.2, svRNA-TFO-E, and svRNA-TFO-nsp3) or separately among SARS-CoV-2-related (svRNA-TFO-nsp2). Phylogenies were rooted using BtKY72 (KY352407.1), except for the E-ORF phylogeny, which was rooted with RmYN05 (MZ081376.1) for visualization purposes. Phylogenetic trees and TFO-like sequence alignments were visualized using *ggtree* (v3.6.2) [88] and *ggmsa* (v1.4.0) [89], respectively.

Genome-wide and ORF-constrained permutation testing with pairwise Wilcoxon rank sum test and FDR multiple test correction [90] was used to test for a nonrandom association between the 6 putative svRNA-TFOs identified in this study and the previously identified recombination breakpoints (*n* = 21) and hotspots of 475 nt length (*n* = 2) in the Wuhan-Hu-1 genome (NC_045512.2) [84]. Base R (v4.2.1) was used to randomly generate 1,200 sites, or the number of sites equal to 10% of the sequence length for the genome-wide and ORF-constrained analysis, respectively. For the ORF-constrained analysis, nearest distance measures were normalized per kilobase (kb) length for each respective region.

### Short BLASTn-based host genome homology search strategy

High homology regions (HHRs) were identified in the genomes of SARS-CoV-1 and SARS-CoV-2 by comparing them to the human reference genome (GRCh38) and genomes of other mammals known to carry diversity of *Sarbecovirus*. This includes the Chinese pangolin (*Manis pentadactyla*), masked palm civet (*Paguma larvata*), and greater horseshoe bat (*Rhinolophus ferrumequinum*) (Table S2). We also examined the genomes of the Malaysian porcupine (*Hystrix brachyura*) and common raccoon dog (*Nyctereutes procyonoides*), which are potential host species linked to SARS-CoV-2-positive PCR samples from the Huanan market in Wuhan, China [91,92]. Chicken (*Gallus gallus*) and San Diego ring-necked snake (*Diadophis punctatus similis*) served as control hosts.

To prepare for BLASTn-based short searching (v2.6.0+) [93], we segmented the reference genomes of SARS-CoV-2 (Wuhan-Hu-1; NC_045512.2) and SARS-CoV-1 (Tor2; AY274119.3) using SeqKit (v0.12.1) [65], with a window length of 25 nt and a single-nucleotide step. We identified hits with 100% identity, an *e*-value threshold of 0.8, a minimum word size of 7, a gap-open penalty of 5, and a gap-extend penalty of 2. We normalized the total hits by the total length in gigabase pair (Gbp) for each animal host genome and visualized the results as a heatmap using ggplot2 (v3.4.4) [78] with a rolling average window size of 20 nt.

### Human enhancer-gene target prediction and enrichment analysis

To identify human enhancer-gene targets that would be most likely affected by the 6 candidate svRNA-TFOs identified in this study, 3 independently measured transcriptomes of differentially expressed genes (DEGs) were interrogated. As mentioned above, these include the top 500 most up- and down-regulated genes in Calu-3 cells and human lungs infected with SARS-CoV-2 [45,58] and the 1,730 most up-regulated genes from whole blood transcriptome of patients with severe COVID-19 compared to healthy donors [59], noting that the Wyler *et al.* dataset [45] was also analyzed in the present study for small RNA-seq RPM coverage described above. The DEGs from these studies (GSE148729, GSE147507, and GSE171110) were accessed through the “COVID-19-related gene sets (2021)” using enrichR [94,95].

Next, the enhancer-linked gene targets predicted for the 6 candidate svRNA-TFOs and 12 remaining TFOs were converted from Ensembl gene IDs (ENSGs) into UniProtKB universal gene identifiers using the UniProt Retrieve/ID mapping tool [96]. The number of correctly matched gene identifiers from the 6 candidate svRNA-TFOs to DEGs was then compared to a background DEG detection rate of 13.5% (4,287 out of 31,698 genes/transcripts across 3 transcriptome studies) using a one-sided Fisher’s exact test. To further evaluate significance, a parametric enrichment analysis was conducted by comparing the observed matches to a randomly generated background, based on 1,000 simulated enhancer-gene target sets of equal size derived from the 12 remaining TFOs, with significance assessed using a standard normal distribution test (*z*-score probability).

### svRNA precursor structural predictions and mutation remodeling

To determine the mostly likely svRNA-TFO precursor RNA structure sequences, consensus among locations for 106 conserved RNA structured regions [97], the 777 miRNA predictions from SARS-CoV-2 Wuhan-Hu-1 (described above), and the regions of small RNA-seq coverage were sought as required. Resultingly, the putative precursor structure of the svRNA-TFO-N was expanded to 150-nt length to accommodate for the extremely high-coverage region upstream of the TFO. Because of the heuristic nature of svRNA-TFO precursor sequence selection, the resultant RNA structural predictions are presented as preliminary models, which require further experimental validation to confirm the exact sequence length or structural elements yet to be determined.

To evaluate potential effects of VoC-associated mutations on svRNA-TFO-related RNA structures, the putative precursor structures corresponding to svRNA-TFO-N and svRNA-TFO-S.1 were remodeled for the G29402U (N:D377Y) and A23403G (S:D614G) mutations, respectively. Optimum MFE represented by Gibbs free energy (Δ*G*) in kcal mol^−1^ was used to predict the secondary structure of the 120-nt-long svRNA-TFO-S.1 precursor (from bases 23,304 to 23,423 in Wuhan-Hu-1) at 37 °C using the Andronescu model [98] assuming GU pairs and a circular molecule, with an additional energy contribution for coaxial stacking of helices using RNAfold on ViennaRNA webserver (v2.6.3) [66]. A similar procedure, but with the Turner model [99] without assuming circular molecule, was used to predict the optimum MFE structure for the svRNA-TFO-N precursor (from bases 29,343 to 29,492 in Wuhan-Hu-1). The MFE of optimal-based structures was visualized using Geneious Prime (v2023.2.1) with the partition function for pairing probabilities and the same parameters described above, and the overall MFE of optimal was calculated.

Next, 3-dimensional (3D) models were generated for the putative svRNA-TFO-N and svRNA-TFO-S.1 precursors using the trRosettaRNA webserver [100] with the RNAfold-generated optimum MFE structure as custom secondary structures. The resultant 3D models were visualized in FirstGlance Jmol (v4.31) [101], including base reliability estimates assigned using the local distance difference test according to the trRosettaRNA definitions of very high, ≥90; high, 70 to 90; medium, 50 to 70; and low, <50.

## Results

### Putative svRNA-TFOs map to SARS-CoV-2 recombination breakpoints and hotspots

The current study focused on identifying svRNA-encoding triplex-forming oligonucleotides (svRNA-TFOs) in SARS-CoV-2 and exploring whether they would have the potential to engage in svRNA-mediated epigenetic interference via triplex-formation against enhancer regions in the host’s genome. TFOs are typically identified from sequences by implementing RNA:DNA:DNA base-pairing algorithms that use canonical triplex-formation rules based on *in vitro* experiments [38]. By using Triplexator [61], we identified 18 TFOs from the SARS-CoV-2 Wuhan-Hu-1 reference genome based on searches for putative TFO target sites within consensus enhancer annotations for human lungs [60] (Table S3). This approach was reasoned based on the findings of Cetin *et al.* and Maldanado *et al.* [20,21] that endogenous TFOs have strong affinity for enhancer regions and that SARS-CoV-2 is a respiratory pathogen known to replicate in, and cause damages to, human lung tissues [102].

**Fig. 1.**
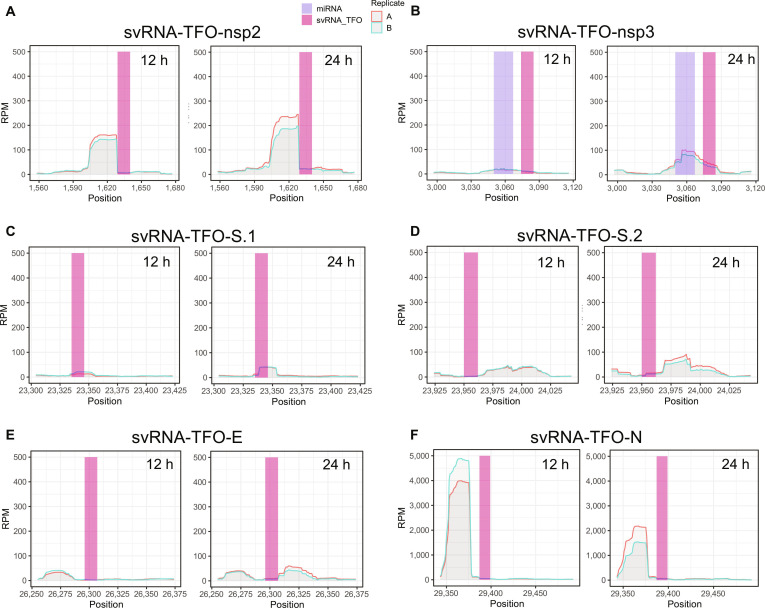
Small RNA-sequencing coverage across genomic regions encoding SARS-CoV-2 small viral RNA (svRNA) triplex-forming oligonucleotides (TFOs). RPM-normalized small RNA-seq coverage is shown for (A) svRNA-TFO-nsp2 (1,558 to 1,677); (B) svRNA-TFO-nsp3 (2,997 to 3,116) and svRNA-nsp3 (SARS-CoV-1) identified by Morales *et al.* [4]; (C) svRNA-TFO-S.1 (23,304 to 23,423); (D) svRNA-TFO-S.2 (23,924 to 24,043); (E) svRNA-TFO-E (26,255 to 26,374); and (F) svRNA-TFN-N (29,343 to 29,492) corresponding to the svRNA-TFO regions marked by numbered arrows and rectangles in Fig. 2A. For the 4-h time points and controls (mock-infected and untreated), see Figs. S2 and S3.

As a proof of concept, the Epi-VIRTEX pipeline considered 446 miRNAs that were predicted in this study from observable SARS-CoV-2 subgenomic RNAs based on nanopore DRS [43] and 331 additional miRNAs predicted from the Wuhan-Hu-1 genome in other studies [69–73] (Table S4). Of the 18 putative TFOs in SARS-CoV-2 described above, 14 were either located adjacent to, or overlapping with, at least one predicted miRNA precursor sequence (Table S3). Next, this information was integrated with RPM-normalized small RNA-seq coverage to confirm infection-relevant signal (≥40 RPM) by 24 hpi within the 25-nt flanks in 6 out of 18 (33%) of the TFOs (Fig. 1A to F). Across the Wuhan-Hu-1 and Tor2 reference genomes, the corresponding 12 hpi small RNA-seq coverage was found to be disproportionately higher over 2 specific regions in the N-ORF and 3′ UTR in both replicates (Fig. S1A). When comparing the distribution of mapped-read lengths for these 2 viruses, SARS-CoV-2 was more consistent between replicates, also noting that reads of 23 to 24 nt length were the most common during the active infection time point at 12 hpi for the Calu-3 small RNA-seq experiment (Fig. S1B and C). Also, little to no level of background coverage was noted from the mock-infected and untreated samples for this dataset, which also demonstrated consistency between A and B replicates libraries, providing confidence in these data (see Figs. S2 to S6).

**Fig. 2.**
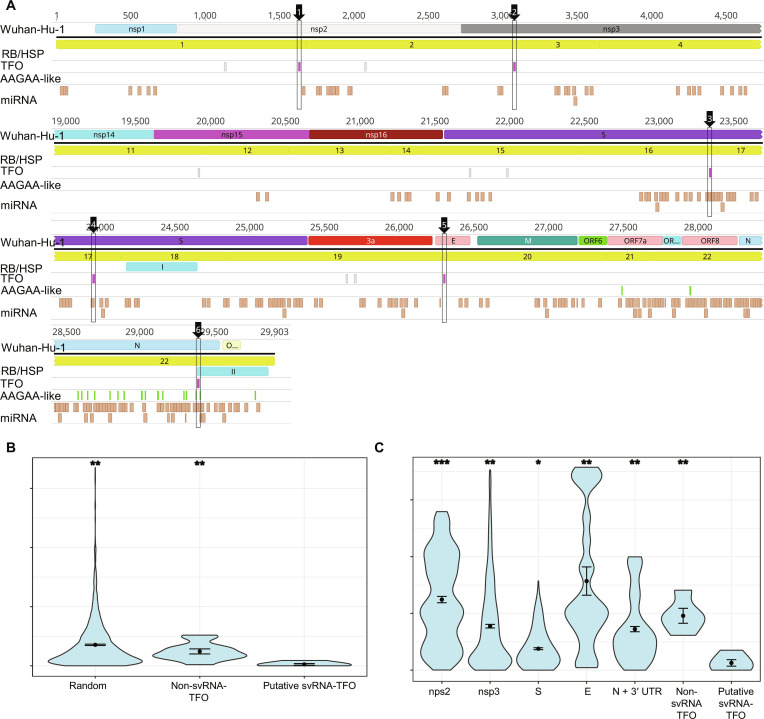
Putative small viral RNA triplex-forming oligonucleotides (svRNA-TFOs) are associated with recombination breakpoints/hotspots in SARS-CoV-2. (A) From top to bottom: (i) Wuhan-Hu-1 reference genome (ASM985889) with main coding regions; (ii) recombination breakpoint (RB) and hotspot (HSP) locations from Lytras *et al.* [84] shown from 1 to 22 in yellow, and I to II in cyan, respectively; (iii) TFOs shown in light gray and pink with the 6 putative svRNA-TFOs of interest indicated by numbered arrows and boxes (see also Fig. 1 and Table S3); (iv) AAGAA-like motif sites reported by Kim *et al.* [43] shown in green; and (v) microRNAs predicted from the observable subgenomic RNAs in this study shown in brown. (B) Nonrandom proximity of RBs and HSPs to ToCs in SARS-CoV-2. Violin plots showing nearest distances of the putative svRNA-TFOs of interest (*n* = 6) and other non-svRNA TFOs (*n* = 12) to actual RBs and HSPs and distances from the putative svRNA-TFOs of interest to randomly generated sites (random). Adjusted (FDR) *P* values: *P < 0.05, **P < 0.01, ***P < 0.001, Wilcoxon rank sum test. Error bars represent standard error of the mean. Genome-wide analysis shows that breakpoints and hotspots are significantly closer to putative svRNA-TFOs than non-svRNA TFOs or random sites. (C) ORF-constrained analysis confirms that breakpoints and hotspots are significantly closer to putative svRNA-TFOs than non-svRNA TFOs and from randomized sites.

These analyses suggest that SARS-CoV-2 produces 6 candidate svRNAs-TFOs, designated as svRNA-TFO-nsp2, svRNA-TFO-nsp3, svRNA-TFO-S.1, svRNA-TFO-S.2, svRNA-TFO-E, and svRNA-TFO-N (Fig. 2A). As previously mentioned, the small RNA-seq coverage in RPM is disproportionately higher for the svRNA-TFO-N compared to other putative svRNA-TFOs identified in this study (Fig. 2 and Fig. S1). Importantly, we found that all 6 of these svRNA-TFOs are located within close proximity (<183 nt) of recombination breakpoints and hotspots previously identified in the Wuhan-Hu-1 reference genome [84] (Fig. 2). A genome-wide analysis determined significant potential for colocalization between the 6 candidate svRNA-TFOs of interest and recombination breakpoints and hotspots (Wilcoxon adjusted [FDR] *P* value = 0.0011). This was determined by comparing the average distance from the nearest recombination breakpoint or hotspot to the candidate svRNA-TFOs (49 nt ± 28 SEM) with the average distance from the remaining 12 non-svRNA TFOs (487 nt ± 85 SEM) and 1,200 randomly generated sites (710 nt ± 23 SEM) (Fig. 2B). Using a similar but more conservative ORF-constrained approach, we observed significant statistical support (Wilcoxon adjusted [FDR] *P* values < 0.05 to < 0.001) that the 6 candidate svRNA-TFOs are located closer to recombination breakpoints and hotspots rather than other non-svRNA TFOs and randomly generated sites (Fig. 2C).

### Enrichment of svRNA-TFO targets across infection-relevant transcriptomes

To identify potential targets of svRNA-mediated epigenetic interference during COVID-19, we identified 120 genes that were differentially expressed upon SARS-CoV-2 infection and which overlapped with the 350 genes associated enhancer regions predicted to be targeted by the 6 candidate svRNA-TFOs (Fig. S7). This corresponds to an approximate 2.5-fold enrichment over the background DEG rate, which is statistically significant (one-sided Fisher’s test, *P* value < 0.001; Fig. S8A). To further test for enrichment, the set of 120 overlapping DEGs was then compared to the non-svRNA TFO-based background simulated from 1,000 random enhancer-target sets, which was also highly significant (*P* value ≈ 0; Fig. S8B). Notably, 16% of the DEG-matched enhancer-gene targets (AHNAK, ALDH4A1, APOBEC3A, CD82, CHAC1, DLL4, EGFL7, FZD4, GAS6, IFNGR2, IL1R2, LILRB4, MYH10, NAPA, PLXNA2, SERPINB1, SPSB1, TNS1, and UGCG) were shared across 2 independent transcriptome studies included in our analysis, highlighting these as potentially the most plausible targets of svRNA-mediated epigenetic interference in lungs during infection of SARS-CoV-2 (Fig. S7 and Table S5).

### Analyses of 6 svRNA-TFOs in SARS-CoV-2 and SARS-CoV-1

**Fig. 3.**
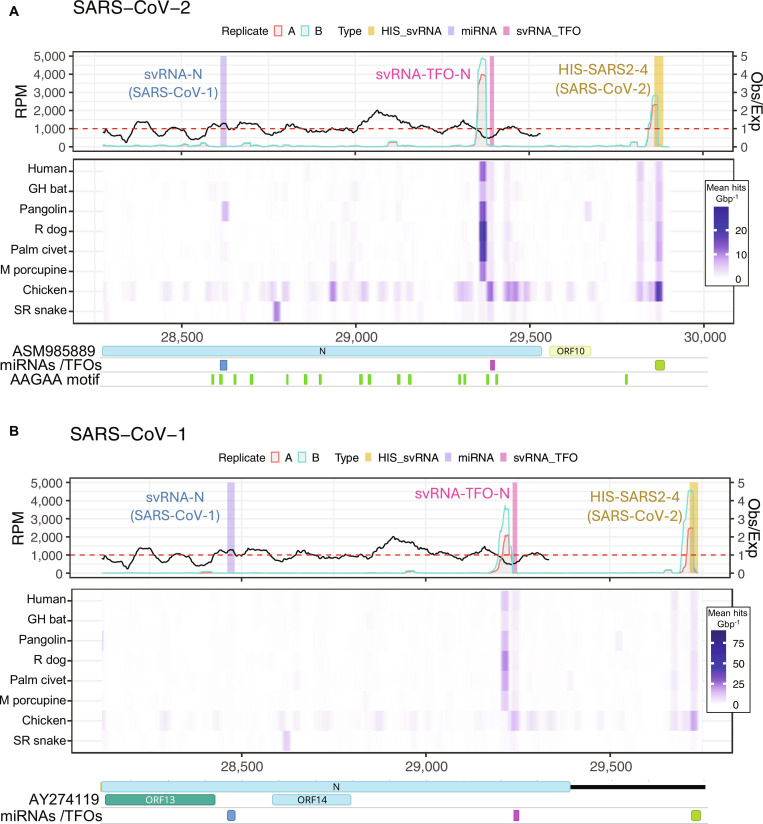
Small RNA-seq, synonymous-site conservation, and host genome homology across the (N)ucleocapsid gene and 3′ UTR. Topmost plots: first *y*-axis: reads per million (RPM) coverage small RNA-seq from Calu-3 cells 12 h postinfection [45]; second *y-*axis: synonymous-site conservation (SSC) analysis for *Sarbecovirus*, red dashed line representing an equal ratio of observed (Obs) synonymous mutations compared to the number of expected (Exp). Middle heatmaps: rolling average BLAST hits against various animal genomes (GH bat = greater horseshoe bat; R dog = raccoon dog; M porcupine = Malaysian porcupine; and SR snake = San Diego ring-necked snake) (see Materials and Methods). Bottom features plots: TFOs predicted against human lung enhancer sequences, including candidate svRNA-TFOs identified using a functional genomics pipeline in this study (Table S3); HIS-SARS2-4 (SARS-CoV-2) was identified by Li *et al.* [7]; “AAGAA-like” (AAGAA) motifs were identified by Kim *et al.* [43]; and svRNA-N (SARS-CoV-1) was identified by Morales *et al.* [4]. (A) SARS-CoV-2 genetic region 28,274 to 29,903 in Wuhan-Hu-1 reference genome. (B) SARS-CoV-1 genetic region 28,120 to 29,750 in Tor2 reference genome.

Of the 6 candidate svRNA-TFOs identified in this study, the most conspicuous is svRNA-TFO-N, which partially overlaps with 1 of 2 dominant small RNA-seq high-coverage regions detected in both SARS-CoV-2 and SARS-CoV-1 (Fig. 3A and B). In SARS-CoV-2, a marked reduction in small RNA-seq coverage is observed at nucleotides 29,376 to 29,492 compared to the region directly upstream (Fig. 3A). Based on small RNA-seq read mapping in the svRNA-TFO-N region, the most frequently mapped start and end locations are at bases 29,352 and 29,376 in the Wuhan-Hu-1 genome, respectively (Fig. S9). The location of the abrupt drop in small RNA-seq coverage overlaps with 2 secondary modification sites, known as “AAGAA-like” motif sites [43], which are located directly upstream and downstream of the TFO. The svRNA-TFO-N encodes a polypurine type TFO motif (5′-AAAAAGAAGAAGG-3′), which is predicted to have binding affinity for the enhancers of genes encoding the CREB protein (CREBBP), alanyl aminopeptidase (ANPEP), interferon stimulated exonuclease gene (ISG20), and frizzled class receptor 4 (FZD4). Expression of these genes was found to be significantly different during infection of SARS-CoV-2 in COVID-19, human lungs, or Calu-3 cells based on previous transcriptome analyses (Fig. S7F).

Using Synplot2 to assess synonymous-site conservation (SSC) among *Sarbecovirus* genomes and MRCA aligned sequences, we observed a pronounced increase in SSC across most of the svRNA-TFO-N region (Fig. 3). Short 25-nt SARS-CoV-2 genome segments were also queried against animal genomes using BLASTn, revealing a discrete and significant (adjusted *P* values < 0.001) overlap between a mammalian-specific HHR and the high-coverage small RNA-seq region corresponding to the same area (Fig. 3A and Table S6). Two additional regions in the 3′ UTR of the Wuhan-Hu-1 reference genome showed relatively high homology to animal genomes, including one HHR associated with the namiRNA HIS-SARS2-4, which partially overlaps the poly-A tail region [7]; these patterns were consistent in both SARS-CoV-2 and SARS-CoV-1 (Fig. 3A and B). The TFO associated with svRNA-TFO-N is a conserved 12-nt polypurine motif (5′-AARAAGAARAAG-3′) (Fig. S10C) present in all *Sarbecovirus* genomes except RsYN04, which contains a cytosine at position 10 (Fig. S10A and B).

To explore effects of mutations associated with SARS-CoV-2 VoCs on putative svRNA-TFO precursor structures, optimum MFE and 3D RNA structure remodeling analyses were used. A 150-nt sequence was used to represent the svRNA-TFO-N precursor from the Wuhan-Hu-1 strain, with a thermodynamically stable optimum MFE structure containing multibranched hairpins (Fig. S11A). The bases that had the most RPM in small RNA-seq coverage (29,343 to 29,376 in Wuhan-Hu-1) were found on an unpaired end within an area that was not predicted with high probability or reliability in either the optimum MFE or 3D structural models, respectively. However, in both models, a higher-confidence hairpin spanning 29,422 to 29,463 was consistently observed (Fig. S11A and B). When comparing the predicted optimum MFE of the svRNA-TFO-N precursor structure from Wuhan-Hu-1 against the one predicted from Delta (B1.617.2), which carries the U29402 (Y377) variant of the D377Y mutation, only a small increase in the Δ*G* from −31.90 to −31.10 kcal mol^−1^ was found (Fig. S11A and C). Similarly, for the 3D structural model, this mutation only had a minor effect on the overall reliability average, which slightly decreased from 65.8 to 64.2 (Fig. S11B and D). Using both the optimum MFE and 3D structural models, the G-to-U transversion at position 29,402 is predicted to confer a change in the loop of a hairpin that is located downstream from the TFO, which is also a site for one of the most common secondary modifications known from SARS-CoV-2 [43] (Fig. S11).

**Fig. 4.**
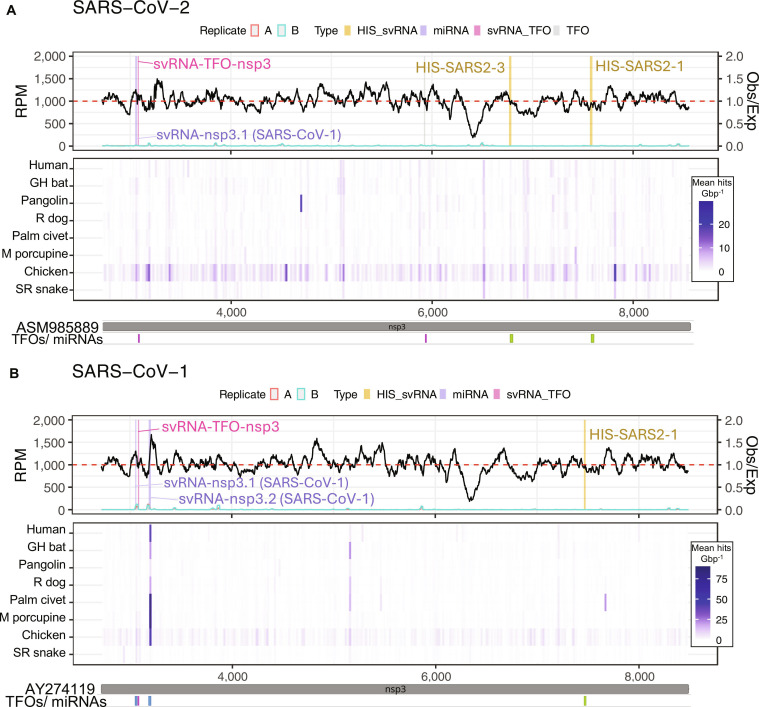
Small RNA-seq, synonymous-site conservation, and host genome homology across the nsp3 region. Topmost plots: first *y*-axis: reads per million (RPM) coverage small RNA-seq from Calu-3 cells 12 h postinfection [45]; second *y*-axis: synonymous-site conservation (SSC) analysis for *Sarbecovirus*, red dashed line representing an equal ratio of observed (Obs) synonymous mutations compared to the number of expected (Exp). Middle heatmaps: rolling average BLAST hits against various animal genomes (GH bat = greater horseshoe bat; R dog = raccoon dog; M porcupine = Malaysian porcupine; and SR snake = San Diego ring-necked snake) (see Materials and Methods). Bottom feature plots: TFOs predicted against human lung enhancer sequences, including candidate svRNA-TFOs identified using a functional genomics pipeline in this study (Table S3); HIS-SARS2-1 and HIS-SARS2-3 were identified by Li *et al*. [7]; svRNA-nsp3.1 (SARS-CoV-1) and svRNA-nsp3.2 (SARS-CoV-1) were identified by Morales *et al.* [4]. (A) SARS-CoV-2 genetic region 2,720 to 8,554 in Wuhan-Hu-1 reference genome. (B) SARS-CoV-1 genetic region 2,719 to 8,484 in Tor2 reference genome.

Beyond the conspicuous svRNA-TFO-N, we also found interesting results in the nsp3 region among SARS-CoV-2 and SARS-CoV-1. When considering small RNA-seq coverage, SSC analysis, and host-homology searches, it was revealed that SARS-CoV-2 and SARS-CoV-1 nsp3 regions both share similar polypurine TFO-like tracts, but strikingly, the small RNA-seq coverage and the presence of an HHR within the vicinity of the TFO are clearly different between the 2 viruses (Fig. 4A and B). The svRNA-TFO-nsp3 in the SARS-CoV-1 encodes a polypurine motif (5′-GAGGAAGAAGAAA-3′), which differs by 3 nts from the svRNA-TFO-nsp3 encoded by SARS-CoV-2 (5′-GAAGAAGAAGAG-3′) (see also Fig. S12). Using short BLASTn, an HHR with significantly higher mean hit counts (*P* values < 0.0001) for several mammalian and chicken genomes was found within the vicinity of a different TFO-like sequence in SARS-CoV-1 (Table S6). This same area also overlaps with a region of high SSC (Fig. 3B), as well as svRNA-nsp3.2 previously characterized by Morales *et al.* [4]. We did not find the same pattern in SARS-CoV-2 (Fig. 4A), with the alignments in this region showing an insertion has occurred in the svRNA-nsp3.2 region among the SARS-CoV-2-related subgroup (Fig. S13). Notably, the svRNA-TFO-nsp3 in SARS-CoV-2 is predicted to interact with only 3 lung enhancer-gene targets, *HADH*, *TMED2*, and *COL16A1*, which are also differentially expressed in Calu-3 cells [45]. Comparatively, this is a much lower number of lung enhancer-gene targets than the other putative svRNA-TFOs identified in this study, which ranged from 6 to 19 (Fig. S7B).

Also, 2 potential triplex-forming svRNAs were identified from the S-ORF regions using the Epi-VIRTEX approach (Fig. S14). svRNA-TFO-S.1 and svRNA-TFO-S.2 were identified as being weakly expressed in Calu-3 cells by SARS-CoV-2 at 24 hpi (Fig. 1C and D) compared to svRNA-TFO-N (Fig. 1F) and svRNA-TFO-nsp2 (Fig. 1A) based on the relative levels of small RNA-seq coverage. The TFOs of svRNA-TFO-S.1 and svRNA-TFO-S.2 are in regions of slightly lower-than-expected SSC, and the TFO of svRNA-TFO-S.1 shares the same location with an HHR specific to some mammals (not Malaysian porcupine) in SARS-CoV-2, but not in SARS-CoV-1 (Fig. S14 and Table S6). The TFO sequence of the svRNA-TFO-S.1 is conserved among *Sarbecovirus*, consisting of a mixed purine-pyrimidine (5′-UUUUGGUGGUGU-3′) motif (Fig. S15A to C). Also, the TFO sequence of svRNA-TFO-S.2 is also a conserved mixed purine-pyrimidine type motif, but its motif contains a palindrome (5′-UUUUGGUGGUUUU-3′) (Fig. S16A to C).

Putative human lung enhancer-gene targets for svRNA-TFO-S.1 include genes for 2 antiviral C-to-U RNA-editing cytidine deaminases APOBEC3A and APOBEC3F, and a nonmuscle myosin heavy chain subcomponent II-B (MYH10) that could be important during infection (Fig. S7C). The enhancer-gene targets of svRNA-TFO-S.2 include several with immune-related functions such as interleukin 1 receptor type 2 (IL1R2), serpin family B member 1 (SERPINB1), and leukocyte immunoglobulin-like receptor subfamily B member 4 (LILRB4) (Fig. S7D).

Using RNA structural remodeling of the S:D614G mutation in the putative svRNA-TFO-S.1 precursor RNA structure, the G23403 variant (characteristic of Alpha [B.1.1.7], Beta [B.1.351], Gamma [P.1], Delta [B.1.617.2], and Omicron [B.1.1.529]) results in a side bulge expansion plus a substantive decrease in the structure’s optimum MFE Δ*G* (−30.19 kcal mol^−1^) when comparing the same structure predicted for Wuhan-Hu-1 (−22.51 kcal mol^−1^) (Fig. S17A and C). The increased stability of the G23403 variant is likely due to the avoidance of an unpaired adenine adjacent to the side bulge. Note that 3D modeling of the putative svRNA-TFO-S.1 precursor resulted in an overall lower reliability model compared to that of the svRNA-TFO-N (Fig. S11B and D); however, the G23403 variant was predicted to exert effects on the orientation of a side bulge similar to the optimum MFE model for the svRNA-TFO-S.1 precursor structure, as well as having effects on the lower-reliability 5′ and 3′ ends (Fig. S17B and D).

While a potentially overlapping HHR to the TFO of svRNA-TFO-E in SARS-CoV-2 and SARS-CoV-1 was detected, but considering the high level of background noise and that the homology of the region was not found to be mammalian-specific, this area most likely represents a false positive and would require further experimental validation. Also, the region shared limited SSC among *Sarbecovirus* compared to the other svRNA-TFOs mentioned above (Fig. S18A and B). The TFO associated with svRNA-TFO-E consists of a 12-nt-long poly-pyrimidine motif (5′-CUUCUUUUUCUU-3′) that is conserved among *Sarbecovirus* (Fig. S19A to C). Potential enhancer-gene targets of svRNA-TFO-E include genes for host cytokine signaling and immune response to viruses, such as IL1R2, interferon gamma receptor 2 (IFNGR2), and tetraspanin CD82 (Fig. S7E). Also, the gene for SNARE-associated NSF attachment protein alpha (NAPA), which is involved in membrane–vesicle fusion, is another potential enhancer-associated target gene of svRNA-TFO-E.

Based on the small RNA-seq coverage in the nsp2 region, SARS-CoV-2 has several areas of >150 RPM coverage compared to SARS-CoV-1, including one high-coverage area adjacent to the TFO from this region (Fig. S20A and B), noting also that the polypurine motif (5′-AAAAAGAGAAAG-3′) is exclusive to the SARS-CoV-2-related subgroup (Fig. S21A to C). Our analysis identified potential enhancer-gene targets for the svRNA-TFO-nsp2 against human lungs, including genes for a growth-arrest specific protein (GAS6), CREBBP, and neuroblast differentiation-associated protein (AHNAK) (Fig. S7A). Lastly, the nsp2 region was not found to contain any major mammal-specific HHRs, and there was limited SSC associated with high-coverage/TFO region (Fig. S20).

By summarizing shared features of interest among the 6 candidate svRNA-TFOs identified in the study, svRNA-TFO-N and svRNA-TFO-S.1 are notably the most plausible for being functional svRNAs; however, these findings would also support svRNA-TFO-nsp2 as a recently emerging svRNA-TFO that is unique to SARS-CoV-2 (Table 1).

**Table 1.** Candidate svRNA-TFO summary of findings

svRNA-TFO	Small RNA-seq coverage	Synonymous-site conservation?	HHR to mammal?	Number of targets	Motif conservation?
svRNA-TFO-nsp2	Medium	No	No	29	Yes, SCV2 group
svRNA-TFO-nsp3	Low	No	No	3	No
svRNA-TFO-S.1	Low	Yes	Yes	23	Yes
svRNA-TFO-S.2	Low	Yes	No	25	Yes
svRNA-TFO-E	Low	Yes	No	18	Yes
svRNA-TFO-N	High	Yes	Yes	32	Yes

## Discussion

Here, using a novel functional genomics approach, we combined analyses of direct sgRNA and small RNA-seq from SARS-CoV-2-infected cells [43,45] with TFO predictions from the Wuhan-Hu-1 reference genome searched against consensus enhancer sequences for human lungs. From this, 6 candidate svRNA-TFOs could be identified for further investigation, including phylogenetics, SSC among *Sarbecovirus*, functional DEG enrichment analysis, and host homology searches, which collectively support epigenetic interference as a plausible virulence mechanism in SARS-CoV-2.

Importantly, all 6 of the candidate svRNA-TFOs identified in this study were significantly colocated within 183 nts of approximately 26% of recombination breakpoints hotspots in the SARS-CoV-2 genome [84]. Comparable patterns have also previously been observed in other viruses. In herpes simplex virus 1, guanine-rich G-quadruplex structures lie within 500 nt of 11% of recombination breakpoints [103,104], and in human immunodeficiency virus, envelope-gene breakpoints consistently colocalize with structured RNA regions and areas of high sequence conservation [105]. While together, these parallels may point toward a broader principle in viral genome biology, namely, that conserved RNA structures and sequence-constrained regions can influence recombination landscape. However, experimental validation is required to confirm whether the connection between the putative svRNA-TFOs and recombination breakpoints and hotspots is causal in SARS-CoV-2. One potential drawback of using analytical-based testing with a general null model is that if the null model is too simple, then the test can result in higher false positives [106]. Nonetheless, several of the candidate svRNA-TFO regions in our study displayed a high degree of SSC across the *Sarbecovirus*, suggesting a deeper evolutionary role for svRNA-TFOs in shaping genome architecture within this lineage.

Unlike traditional miRNA prediction pipelines, which typically predict precursor hairpin structures from full genome sequences, Epi-VIRTEX uses nanopore-based DRS containing the diversity of sgRNAs produced by Wuhan-Hu-1 [43] as a starting point for precursor hairpin prediction. While this approach yields a higher number of mature miRNA predictions than traditional pipelines, this is largely attributed to the high level of redundancy present within the underlying sgRNA-derived sequencing data. To improve specificity, the HuntMi ML algorithm was employed, which incorporates a virus-specific training set for improved detection of viral precursor hairpins [67].

Because Epi-VIRTEX was designed to explore triplex-forming interactions, yet the exact characteristics of svRNA-TFOs remain unknown, we re-analyzed an adapter ligation-free small RNA-seq dataset from SARS-CoV-2-infected Calu-3 cells at 4, 12, and 24 h [45]. This approach is better suited for capturing diverse classes of small RNAs rather than canonical miRNAs, which are typically ~22 nt in length. While canonical miRNAs are the most extensively studied small RNAs, several noncanonical small RNA classes have recently been identified during SARS-CoV-2 infection. These include miRNA-like small RNAs that selectively repress host genes and range in size from 18 to 30 nts [5]. In another study combining Argonaute immunoprecipitation with small RNA-seq, SARS-CoV-2 infection was also shown to alter host-derived 5′ isomiRs, particularly at 24 hpi [107]. In addition, several circRNAs have been recently identified in both SARS-CoV-2 and SARS-CoV-1 [108,109]. Given the diversity of small RNAs now known to comprise the SARS-CoV-2 miRNAome, consistently greater capture of 23- to 24-nt-long small RNAs from SARS-CoV-2-infected Calu-3 cells at 12 hpi is plausible for a new class of svRNA. This is particularly relevant because svRNAs remain critically understudied and currently lack dedicated detection methods and prediction tools.

Thus, while the 6 candidate svRNA-TFOs have only just been revealed through this study, others have already characterized several different triplex-forming long ncRNAs in humans. For example, PARTICLE, MEG3, and Khps1 encode either 1, 2, or 3 TFOs, which can range in length from 18 to 22 nts [26–28]. Also, while PARTICLE and Khps1 are known to be *cis* regulators of genes located directly downstream, MEG3 can act from a distance in *trans*, which is the same mechanism being proposed for the svRNA-TFOs as part of the epigenetic interference hypothesis.

Additionally, high homology between regions of the SARS-CoV-2 and human genomes has been described before as well [7,110], including with the functional characterization of 5 human identical sequence (HIS) namiRNAs produced by SARS-CoV-2 that bind to enhancer sequences and control host gene expression [7]. Our study goes a step further by expanding virus-to-host homology searches against the reference genomes of various mammalian hosts and nonmammalian controls. Strikingly, we show discrete regions of significantly higher-than-average homology specific to mammalian host genomes for svRNA-TFO-N and svRNA-TFO-S.1, which also overlap regions with small RNA-seq coverage and high SSC. While the latter was specific to SARS-CoV-2, another discrete HHR with a less clear host-matching pattern was identified in the nsp3 region of SARS-CoV-1 and must be experimentally validated first. Together, these results suggest a potential role for the epigenetic interference hypothesis in host adaptation in *Sarbecovirus*, which requires further attention.

In another SARS-CoV-2 RNA-based sequencing study, researchers used *in situ* conformation sequencing to map viral genome structure [111]. These results aligned with our model that the A23403G (S:D614G) mutation potentially stabilizes the svRNA-TFO-S.1 precursor RNA structure by creating a more thermodynamically favorable 6-nt side bulge located 56 nt downstream of the TFO motif. This prediction was also consistent with strong SHAPE-MaP signals observed in infected cells [112]. However, beyond its predicted effects on RNA structure, the G614 spike variant is also well known to increase infectivity [113], transmissibility [114], and virion stability [115]. Together, these findings support the possibility that G23403 provided dual advantages by improving both spike protein function and svRNA-TFO-S.1 precursor stability at a pivotal stage of early SARS-CoV-2 evolution. This is consistent with G23403 rising to fixation during an 8-month period of evolutionary stasis beginning in April 2020 and persisting across all subsequent lineages that began diverging in August 2021 [116].

Note that there is already substantial evidence that the G614 spike variant and other nonsynonymous mutations were strongly selected because of their effects at the protein level. For example, Hasan *et al.* [117] characterized the leading mutations in the SARS-CoV-2 spike protein and found that variant of concern-defining mutations were frequently located on the protein surface. Nevertheless, RNA structures may also contribute to selection. In another study, mutation rates across the SARS-CoV-2 genome were reduced at bases involved in RNA base-pairing, suggesting that disruption of RNA structure can have detrimental effects on viral fitness [118].

Furthermore, while these RNA structural models should be interpreted with caution in the absence of experimental data, a second mutation in the N-ORF region, G29402U (N:D377Y), associated with the Delta variant also showed marginal RNA-structural effects, potentially altering a stem loop in the optimum MFE structure and producing subtle effects in the corresponding 3D model. G29402U co-occurs with G28881U (N:R203M), and together, these mutations enhance viral suppression of RIG-I-mediated antiviral signaling [119]. The G-to-U transversion at 28,881 additionally introduces a premature AUG (Met) codon into the nucleocapsid sgRNA located ~500 nt upstream from the start of the svRNA-TFO-N region. This suggests that both G29402U and G28881U may influence svRNA-TFO-N processing, although this requires further confirmation with small RNA-seq comparing svRNA-TFO-N production levels between the Delta variant and Wuhan-Hu-1, which is not currently available at this time.

Notably, A23403G (S:D614G) is consistently accompanied by 3 further mutations (C241U, C3037U, and C14408U) [111], all of which are C-to-U transition, which are known to be the most dominant mutation in SARS-CoV-2 [118]; intriguingly, svRNA-TFO-S.1 is predicted to target enhancer regions of *APOBEC3A* and *APOBEC3F*, which are genes for cytidine deaminases that drive C-to-U conversions and contribute to the known C-to-U mutational bias in SARS-CoV-2 [120,121]. This raises the possibility that svRNA-TFO-S.1 could have been positively selected if APOBEC up-regulation increased the emergence of novel RNase L cut-sites, an idea consistent with the central role of RNase L in SARS-CoV-2 [53–56] and its preference for unpaired UU or UA dinucleotide in stem-loop bulges [122,123]. Supporting this model, Cao *et al.* [111] found that all 3 accompanying mutations (C241U, C3037U, and C14408U) introduce new unpaired UU dinucleotides specifically into bulge regions across 3 distinct stem-loop structures, offering a mechanistic link between APOBEC-driven editing, svRNA-TFO function, and structural evolution of the SARS-CoV-2 genome.

Another major highlight of this study is that, beyond the 6 candidate svRNA-TFOs identified in SARS-CoV-2, 2 previously characterized svRNAs, a canonical miRNA (svRNA_nsp3.2) from SARS-CoV-1 [4] and a namiRNA (HIS-SARS2-4) from SARS-CoV-2 [7], also appear plausibly linked to epigenetic interference. Intriguingly, the nsp3 region in both SARS-CoV-1 and SARS-CoV-2 contains multiple TFO-like polypurine motifs that cluster near a known recombination breakpoint [84], strongly suggesting that this locus has an evolutionary history involving recombination across the *Sarbecovirus* lineage. Our comparative analysis of SARS-CoV-2 and SARS-CoV-1 indicates that svRNA-nsp3.2 may participate in epigenetic interference in only SARS-CoV-1, as it overlaps one of these TFO-like motifs, is produced at high abundance in Calu-3 cells, and has an HHR against several mammalian host genomes unique to the Tor2 reference genome. Together, these observations point to a broader network of svRNAs, including high-coverage svRNA-TFO-N, svRNA-nsp3.2, HIS-SARS2-4, and possibly the other 5 candidate svRNA-TFOs identified here. Potentially, these svRNAs may act synergistically in epigenetic interference, an area that warrants dedicated experimental investigation.

### Limitations

While application of advanced RNA-seq technologies on SARS-CoV-2 has created unprecedented opportunities to interrogate virus evolution as demonstrated in this study, the proposed mechanism of svRNA-mediated epigenetic interference remains hypothetical and requires experiment validation before it can be accepted as a recognized virulence pathway in COVID-19. Although Epi-VIRTEX was designed for conservative capture of putative svRNA-TFOs based on sufficient small RNA-seq coverage, the bioinformatics prediction of miRNAs from the SARS-CoV-2 sgRNA likely increases the number of miRNA predictions because of both a larger number of starting sequences and a permissive optimum MFE cutoff of 20 kcal mol^−1^. Therefore, additional experimental validation of viral-derived svRNA-TFOs is needed. Particularly valuable would be studies examining RNA stability, nuclear localization, and sequence-specific heterotriplex formation at predicted enhancer-gene targets. Recent advances in computational chemistry have enabled modeling of SARS-CoV-2 protein interactions and evolution through molecular dynamics (MD) simulations [124–126]. However, major technical limitations remain for MD simulations of RNA:DNA:DNA triplexes [22]. For svRNAs-TFOs, future modeling efforts could be greatly informed by additional SHAPE-MaP data for svRNA precursor structures and structural studies of RNA:DNA:DNA triplexes using crystallography. Other validation approaches may include electromobility shift assays to demonstrate sequence-specific RNA binding of candidate svRNA-TFOs to enhancer-gene targets, or triplex capture methods involving chromatin RNA immunoprecipitation.

Importantly, it also remains necessary to confirm that small RNA-seq coverage reflects genuine svRNA-TFO production rather than background RNA degradation from highly transcribed genomic regions. In SARS-CoV-2, the N-ORF and 3′ UTR are the most highly transcribed regions of the Wuhan-Hu-1 genome [43], and these regions overlap with the only 2 high-coverage regions detected in the small RNA-seq from Calu-3 cells at 12 and 24 hpi. Although this study did not explicitly characterize the exact sequences, sizes, or the 5′-/3′-end chemistry of these RNAs, we found evidence of precise 3′-end clustering within the svRNA-TFO-N region consistent with defined processing. Furthermore, the high-coverage signals were tightly localized and conserved between the Wuhan-Hu-1 and Tor2 reference genomes. These findings are supported by independent evidence verifying expression of both svRNA-TFO-N and the high-coverage 3′ UTR in A549 ACE2-cells by quantitative reverse transcription PCR [110]. Notably, the high-coverage 3′ UTR also encodes the namiRNA HIS-SARS2-4, which regulates expression of *hyaluronan synthase 2* during COVID-19 [7]. The close proximity and relatively high coverage of HIS-SARS2-4 and svRNA-TFO-N (within ~550 nt of one another) suggest a shared processing mechanism for namiRNAs and svRNA-TFOs. In contrast, the remaining 5 svRNA-TFOs identified in this study exhibited substantially lower coverage, near the ≥40 RPM threshold, and therefore require further experimental validation to confirm their production and functionality during COVID-19.

Additional limitations of this current study include the narrow focus on datasets relevant to SARS-CoV-2 infection of human lungs and lung-derived cell types and tissue as a biologically grounded starting point. This limitation was addressed by the utilization of 3 previously published transcriptome studies involving SARS-CoV-2 infections of Calu-3 cells, undifferentiated human bronchial epithelial cells, and whole blood of severe COVID-19 patients. Additional research extending these analyses to other important infection-relevant cell types and tissues, such as immune cells for example, would together with direct molecular evidence also help to define the broader aspects of the putative epigenetic interference mechanism. While many datasets remain available for secondary and higher-order functional analyses in SARS-CoV-2, reliance on previous published studies limited the ability to determine whether svRNA-TFO enhancer-gene targets are specifically dysregulated during COVID-19 or reflect broader lung infection responses, and further experimental validation is needed to confirm these results.

## Conclusion

By integrating RNA-seq data, evolutionary models, and sequence analysis, we propose that candidate svRNA-mediated epigenetic interference may provide further insights into the evolution of SARS-CoV-2 and SARS-CoV-1 within the *Sarbecovirus* lineage. In total, we identified 6 candidate svRNA-TFOs in SARS-CoV-2, with 2 of these (svRNA-TFO-N and svRNA-TFO-nsp2) produced at high abundance as early as 4 hpi in Calu-3 cells. While production and functionality of the 6 candidate svRNA-TFOs remain to be experimentally validated, we confirmed our predictions of functional svRNA-TFO enhancer-gene target relationships in human lung tissue by showing significant enrichment of enhancer-gene targets among human DEGs detected across 3 independent transcriptome studies. These findings, along with recent support from another study confirming that svRNA-TFO-N is also highly expressed in A549 ACE2-cells [110], strongly support the epigenetic interference hypothesis as a viable mechanism in SARS-CoV-2.

Furthermore, our predictive structural modeling of the svRNA precursors for svRNA-TFO-N and svRNA-TFO-S.1 suggests a potential link to respective mutations that became characteristic in later SARS-CoV-2 VoCs, including S:D614G, and N:D377Y. While much further work is required to fully validate the epigenetic interference hypothesis in SARS-CoV-2, including further validation of the mammalian-specific homologies identified for svRNA-TFO-N and svRNA-TFO-S.1, such efforts are likely to refine our understanding of the natural drivers of *Sarbecovirus* evolution and inspire novel antiviral strategies. Ultimately, these advances may enhance preparedness for potential future outbreaks of SARS-CoV-like viruses, which continue to pose major threats to human health and global stability.

## Data Availability

No new datasets were generated as a result of this study. All datasets supporting the conclusions of this article are referenced within the article and its additional files. To analyze these data, Epi-VIRTEX was developed, which includes open-access codes supporting the conclusions of this study. See the Epi-VIRTEX homepage for more information at https://github.com/damselflywingz/EpiVIRTEX. An archived version of the code was deposited here: https://doi.org/10.5281/zenodo.18346114.
