# Supplementary material for: Functional Genomic Evidence for Candidate Small Viral RNA-Mediated Epigenetic Interference in SARS-CoV-1 and SARS-CoV-2
Source: Comput Struct Biotechnol J. 2026 Jul 2;35(1):0148. doi: 10.34133/csbj.0148 (PMC13323714; doi:10.34133/csbj.0148)
Supplement: Supplementary 1 — Tables S1 to S6 Figs. S1 to S21 [file csbj.0148.f1.zip › 20260504_Supplemental_information_PaulsonMontoyaJoy2026.pdf]

Supplemental information

Paulson, A.R., Montoya V., and Joy, J.B. 2026. Functional genomic evidence for candidate small viral RNA-mediated epigenetic interference in SARS-CoV-1 and SARS-CoV-2.

List of Tables and Figures

Supplemental Figure S1. 5
Supplemental Figure S2. 6
Supplemental Figure S3. 7
Supplemental Figure S4. 8
Supplemental Figure S5. 9
Supplemental Figure S6. 10
Supplemental Figure S7. 11
Supplemental Figure S8. 14
Supplemental Figure S9. 15
Supplemental Figure S10. 16
Supplemental Figure S11. 17
Supplemental Figure S12. 18
Supplemental Figure S13. 19
Supplemental Figure S14. 20
Supplemental Figure S15. 21
Supplemental Figure S16. 22
Supplemental Figure S17. 24
Supplemental Figure S18. 25
Supplemental Figure S19. 26

Supplemental Table S1. 2
Supplemental Table S2. 2
Supplemental Table S3. 3
Supplemental Table S4. 3
Supplemental Table S5. 11
Supplemental Table S6. 13

References 27

Supplemental Table S1. Sources and accession numbers for SARS-related coronavirus genomes used for phylogenetic reconstruction and synonymous-site conservation analysis for nsp2, nsp3, S-, E-, and N-ORF regions.

20260329_Supplemental_Table_S1.xlsx

Supplemental Table S2. Animal host genome resources used in this study.

20260329_Supplemental_Table_S2.xlsx

Supplemental Table S3. Detection of triplex-forming oligonucleotides (TFOs) of concern in SARS-CoV-2 using microRNA (miRNA) predictions and small RNA-sequencing (RNA-seq)*.

TFO position	# Triplex target sites	TFO type	Open reading frame	Overlap with any predicted miRNA precursor (see supplemental Table S4)?	≥ 40 RPM Small RNA-seq coverage within 25 nt of TFO?
1,130 – 1,141	79	GA	nsp2	Kahn et al. 2020	No
1,629 – 1,640	48	GA	nsp2	This study (adjacent)	Yes
2,072 – 2,083	91	GU	nsp2	Kahn et al. 2020; Liu et al. 2021	No
3,074 – 3,085	7	GA	nsp3	Saini et al. 2023	Yes
5,920 – 5,932	133	GU	nsp3		No
9,247 – 9,258	106	GU	nsp4	This study; Kahn et al. 2020	No
11,174 – 11,187	527	GU	nsp6	This study	No
11,795 – 11,810	635	GU	nsp6	Liu et al. 2021	No
15,726 – 15,740	80	GU	nsp12	Kahn et al. 2020	No
19,916 – 19,927	110	GU	nsp15		No
21,731 – 21,742	38	CU	S	This study	No
21,980 – 21,992	97	GU	S	Kahn et al. 2020	No
23,335 – 23,346	81	GU	S	This study	Yes
23,950 – 23,962	160	GU	S	This study	Yes
25,643 – 25,655	102	GU	ORF3a		No
25,700 – 25,711	130	CU	ORF3a		No
26,296 – 26,307	53	CU	E	This study; Kahn et al. 2020	Yes
29,387 – 29,399	101	GA	N	This study	Yes

*Small RNA-seq data from SARS-CoV-2 during infection of Calu-3 cells measured in reads per million (RPM), see Wyler *et al.* 2021 [1].

Supplemental Table S4. Predicted microRNA annotations for SARS-CoV-2 Wuhan-Hu-1 used in this study. See Materials and Methods for more information about the referenced studies.

20260329_Supplemental_Table_S4.xlsx

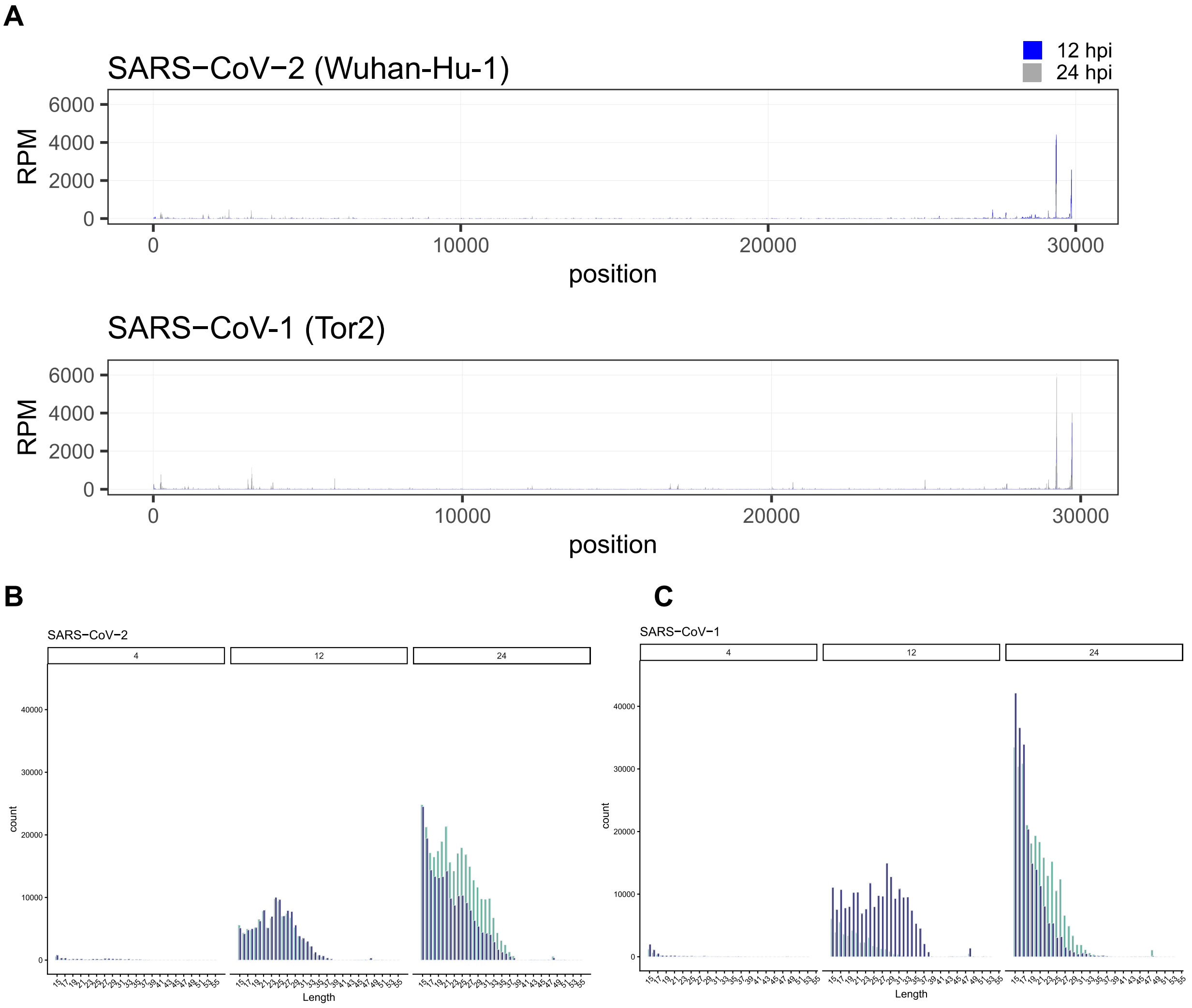

Supplemental Figure S1. Genome-wide small RNA-seq coverage in reads per million (RPM) and mapped-read length distributions. A) Average genome-wide coverage from small RNA-seq collected in duplicate at 24 and 12 hpi of Calu-3 cells for SARS-CoV-2 (top) and SARS-CoV-1 (bottom). **B)** Mapped-read length distribution from duplicate small RNA-seq collected from Calu-3 cells at 4, 12, and 24 hpi with SARS-CoV-2. C) Same as B, but for SARSCoV-1. Original dataset collected by Wyler et al. 2021 [1].

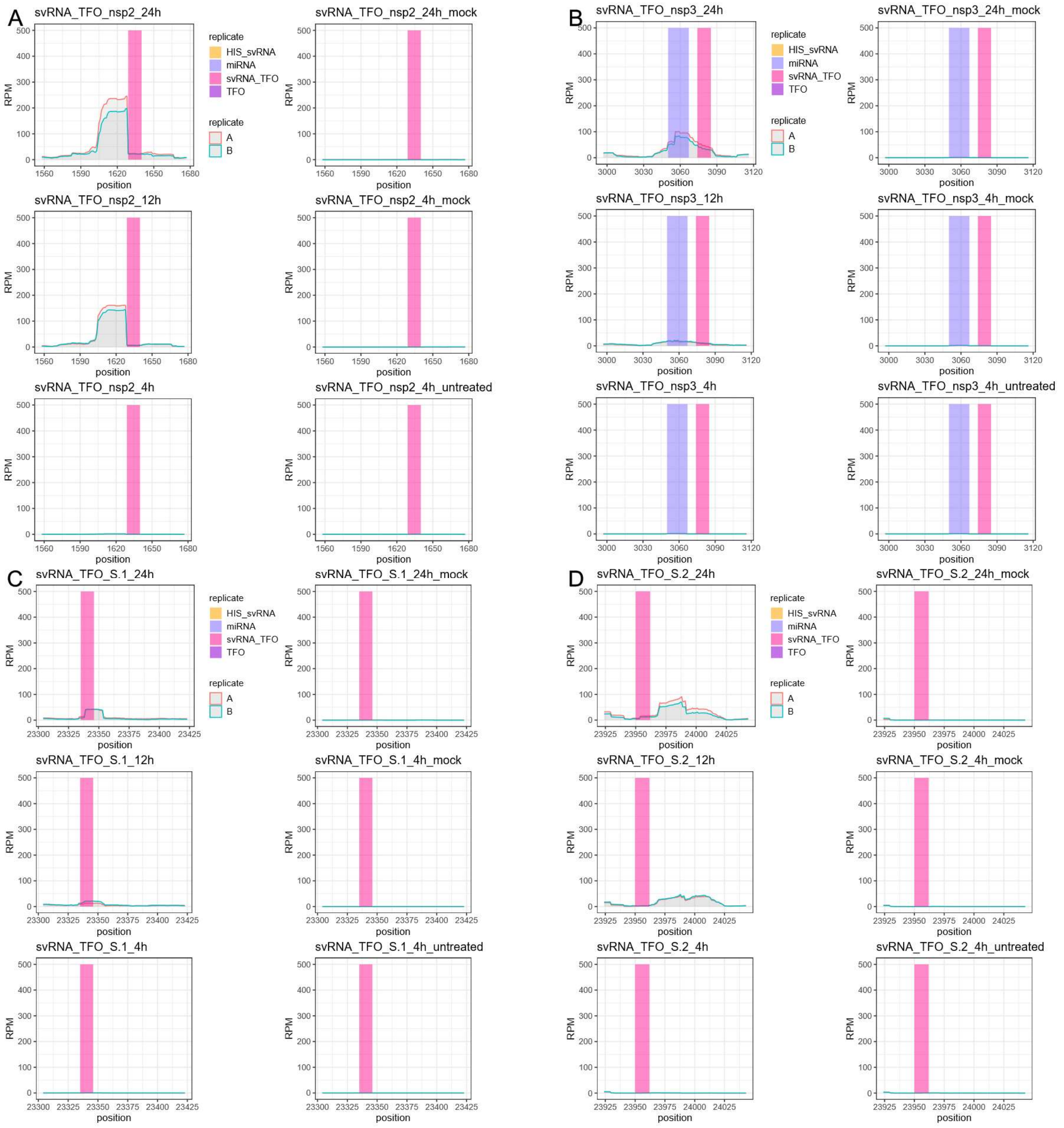

Supplemental Figure S2. Single-nucleotide coverage of small RNA-sequencing reads (A and B replicate) from SARS-CoV-2-infected Calu-3 cells at 24, 12, and 4 hpi, with mock-infected and untreated as controls [1]. **A)** portion of the nsp2 ORF region (1,558 – 1,677) encoding predicted svRNA-TFO-nsp2. **B)** portion of nsp3 ORF region (2,997 –3,116) encoding predicted svRNA-TFO-nsp3. **C)** portion of spike ORF region (23,304 – 23,423) encoding predicted svRNA-TFO-S.1. **D)** portion of spike ORF region (23,924 – 24,043) encoding predicted svRNA-TFO-S.2. The y-axis is presented as the number of reads per million (RPM) and all positions are relative to the Wuhan-Hu-1 genome.

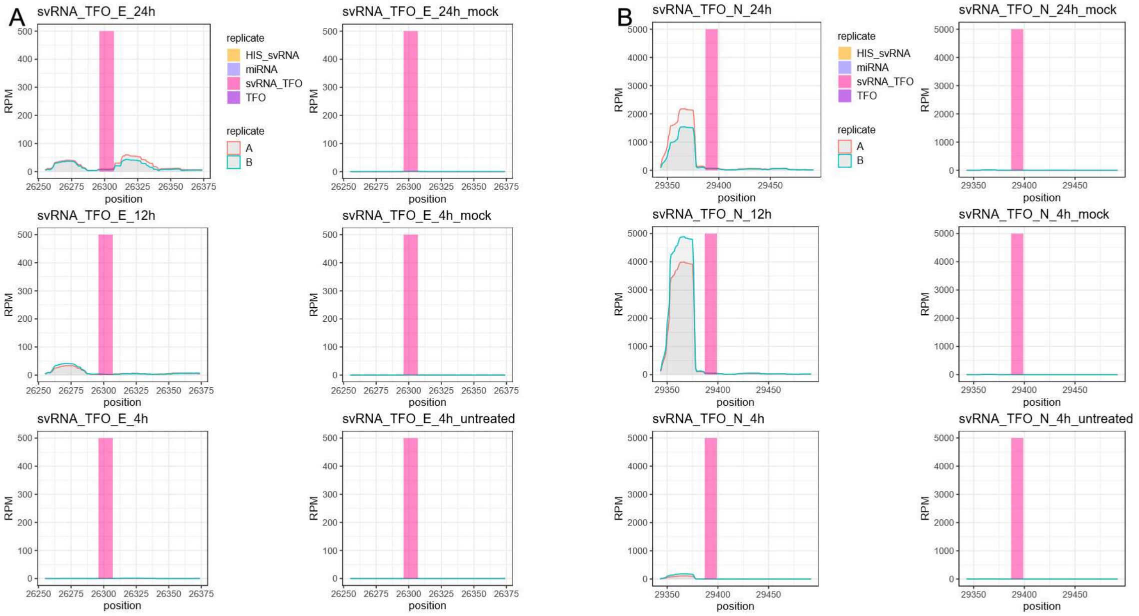

Supplemental Figure S3. Single-nucleotide coverage of small RNA-sequencing reads (A and B replicate) from SARS-CoV-2-infected Calu-3 cells at 24, 12, and 4 hpi, with mock-infected and untreated as controls [1]. **A)** portion of the envelope ORF region (26,255 – 26,374) encoding predicted svRNA-TFO-E. **B)** portion of the nucleocapsid ORF region (29,343 – 29,492) encoding predicted svRNA-TFO-N. The y-axis is presented as the number of reads per million (RPM) and all positions are relative to the Wuhan-Hu-1 genome.

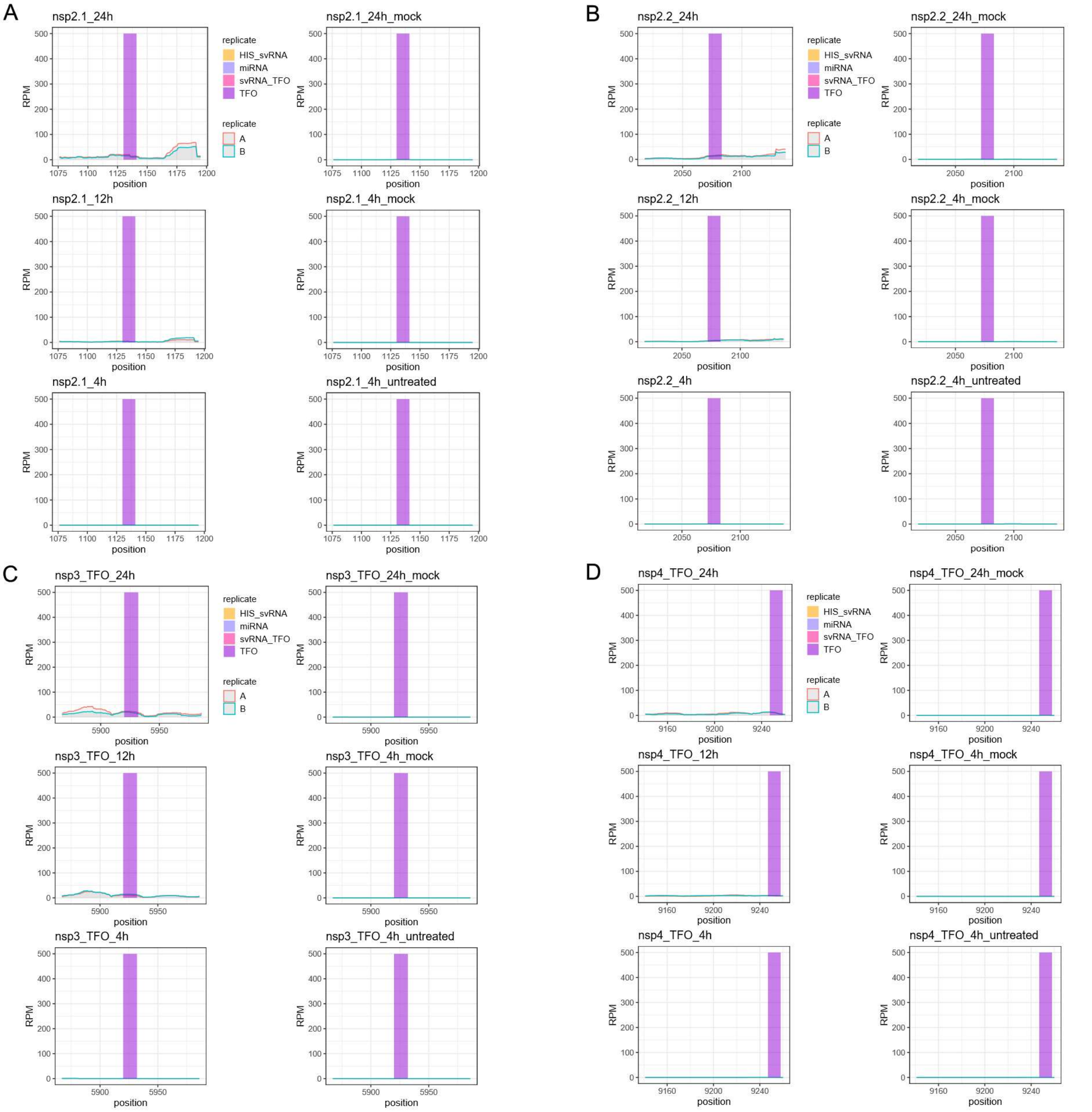

Supplemental Figure S4. Single-nucleotide coverage of small RNA-sequencing reads (A and B replicate) from SARS-CoV-2-infected Calu-3 cells at 24, 12, and 4 hpi, with mock-infected and untreated as controls [1]. TFO encoding regions: **A)** portion of the nsp2 ORF region (1,076 – 1,195). **B)** portion of nsp2 ORF region (2,018 – 2,137). **C)** portion of nsp3 ORF region (5,867 – 5,986). **D)** portion of nsp4 ORF region (9,141 – 9,260). The y-axis is presented as the number of reads per million (RPM) and all positions are relative to the Wuhan-Hu-1 genome.

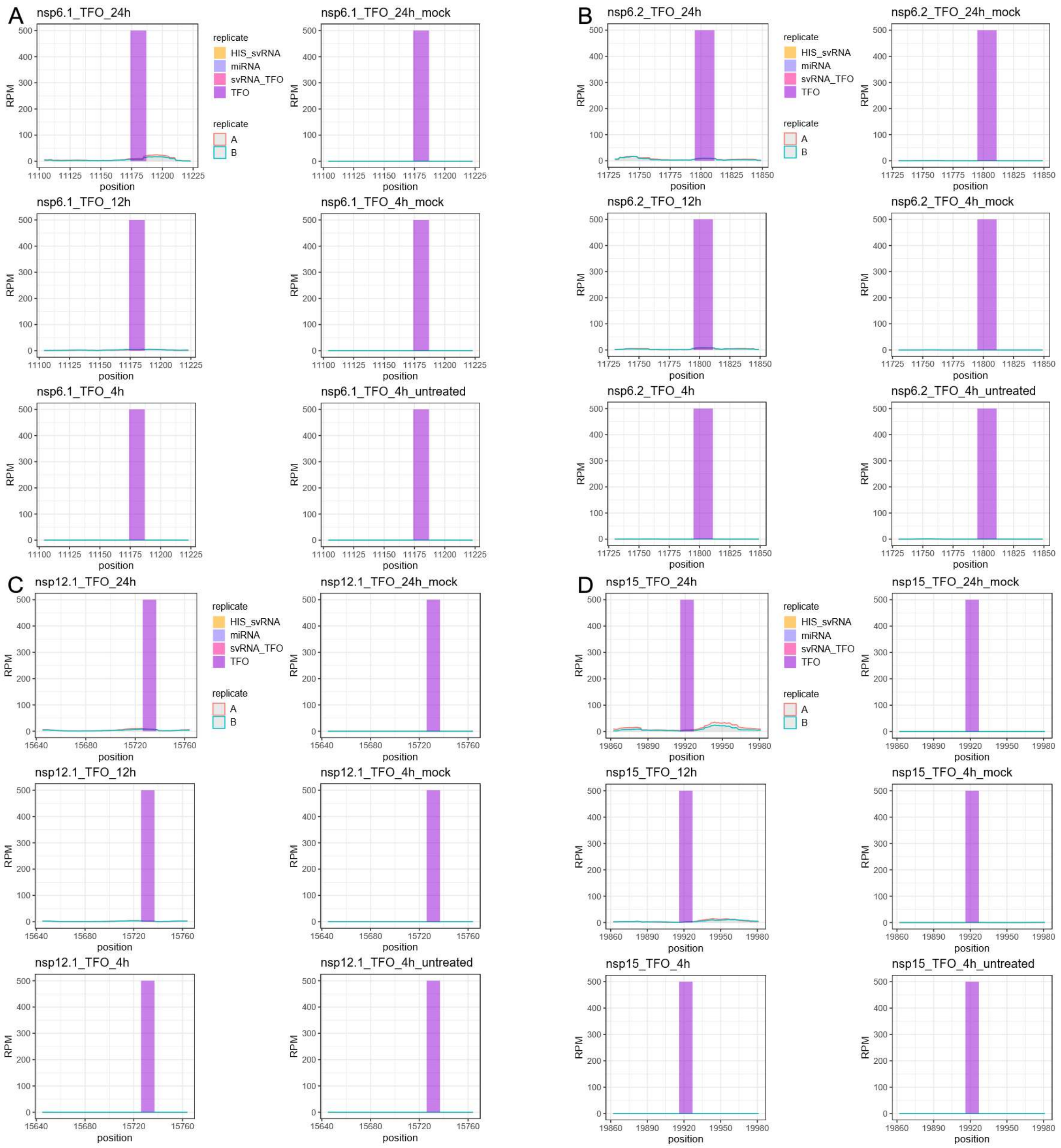

Supplemental Figure S5. Single-nucleotide coverage of small RNA-sequencing reads (A and B replicate) from SARS-CoV-2-infected Calu-3 cells at 24, 12, and 4 hpi, with mock-infected and untreated as controls [1]. TFO encoding regions: **A)** portion of the nsp6 ORF region (11,104 – 11,223). **B)** portion of nsp6 ORF region (11,730 – 11,849). **C)** portion of nsp12 ORF region (15,645 – 15,764). **D)** portion of nsp15 ORF region (19,862 – 19,981). The y-axis is presented as the number of reads per million (RPM) and all positions are relative to the Wuhan-Hu-1 genome.

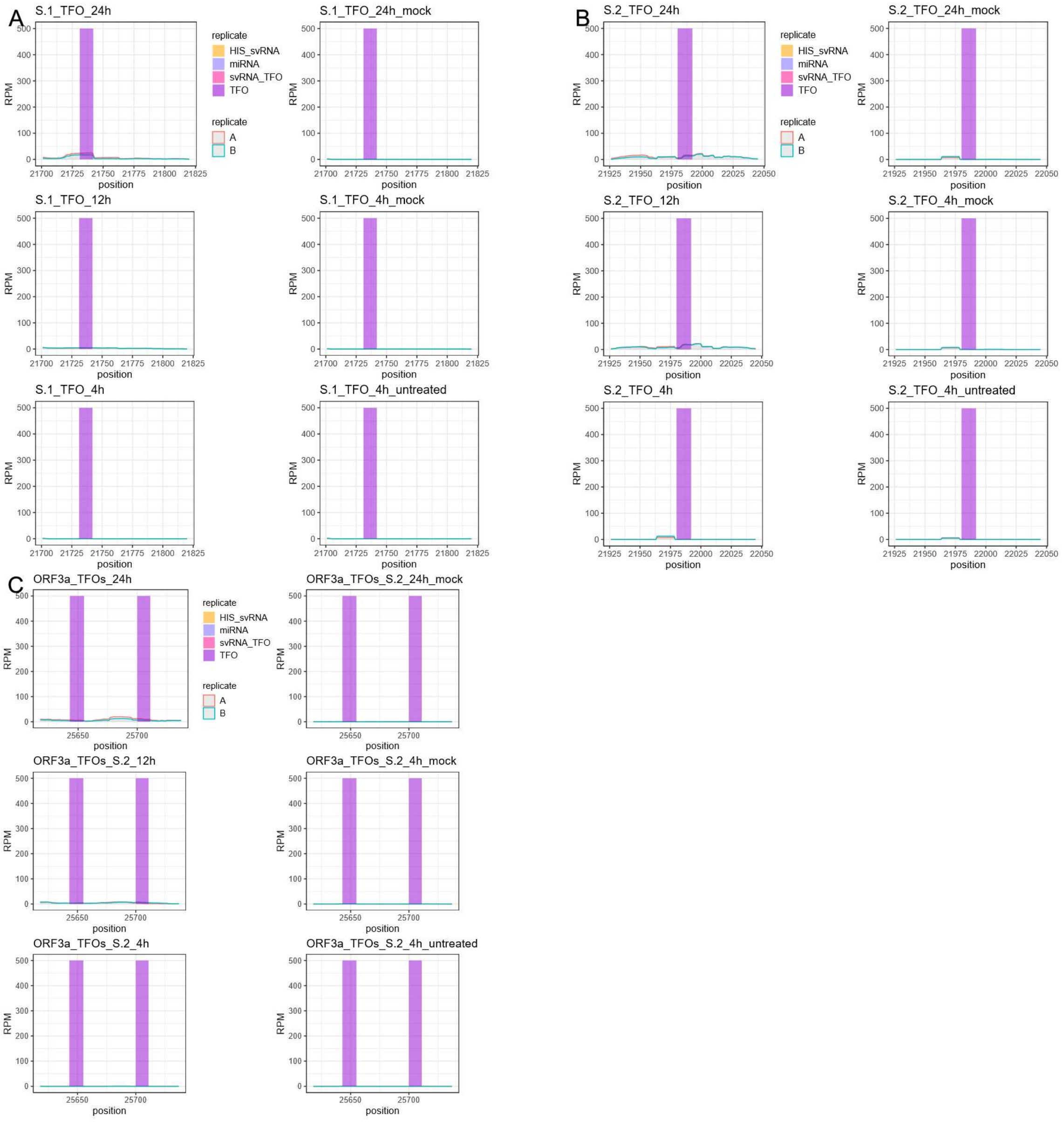

Supplemental Figure S6. Single-nucleotide coverage of small RNA-sequencing reads (A and B replicate) from SARS-CoV-2-infected Calu-3 cells at 24, 12, and 4 hpi, with mock-infected and untreated as controls [1]. TFO encoding regions: A) portion of the spike ORF region (21,701 – 21,820). **B)** portion of spike ORF region (21,926 – 22,045). C) portion of ORF3a region (25,618 – 25,737). The y-axis is presented as the number of reads per million (RPM) and all positions are relative to the Wuhan-Hu-1 genome.

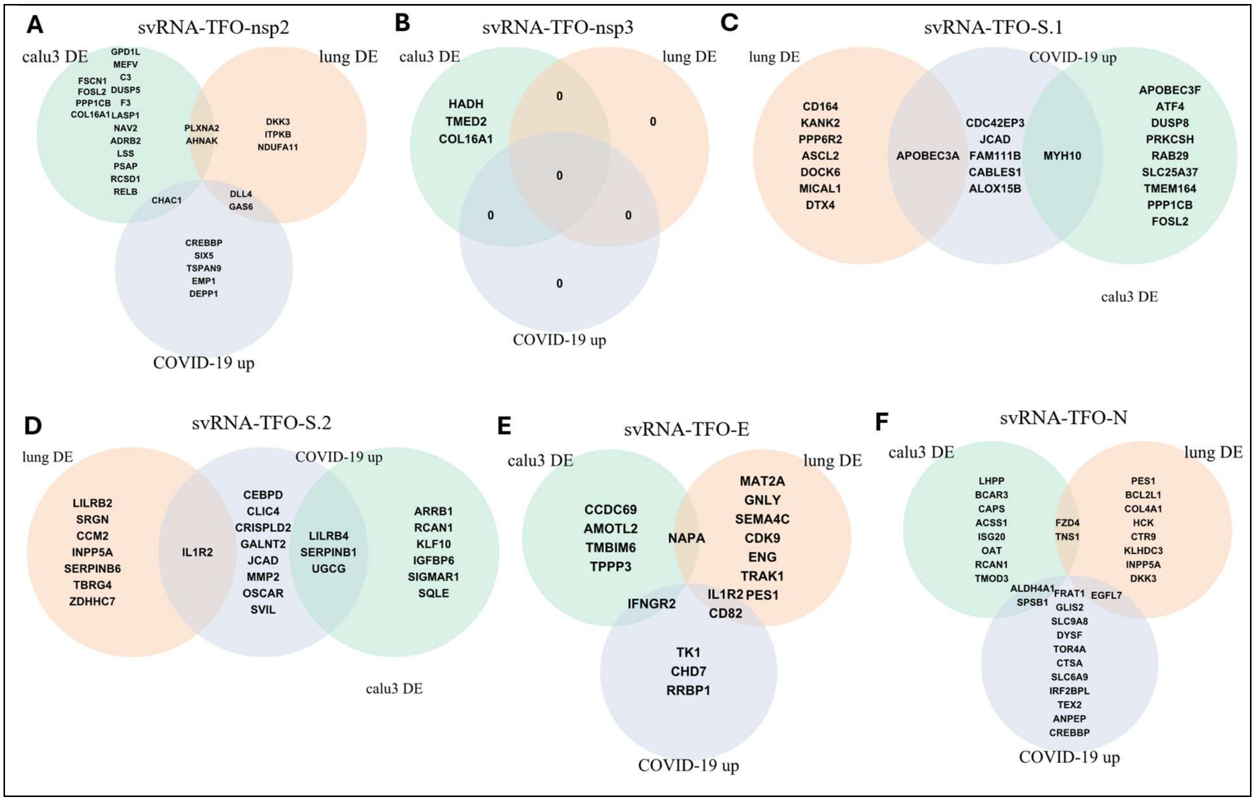

Supplemental Figure S7. Venn diagrams identifying the most likely human lung enhancer-gene targets of the small viral RNA (svRNA) triplex-forming oligonucleotides (TFOs) of concern. Predicted enhancer-gene targets of A) svRNA-TFO-nsp2; **B)** svRNA-TFO-nsp3; **C)** svRNA-TFO-S.1; **D)** svRNA-TFO-S.2; **E)** svRNA-TFO-E; and **F)** svRNA-TFO-N. This analysis was based on the topmost differentially expressed (DE) genes in response to SARS-CoV-2 infection of Calu-3 cells, lungs [1,2] and whole blood transcriptome of patients with severe COVID-19 [3].

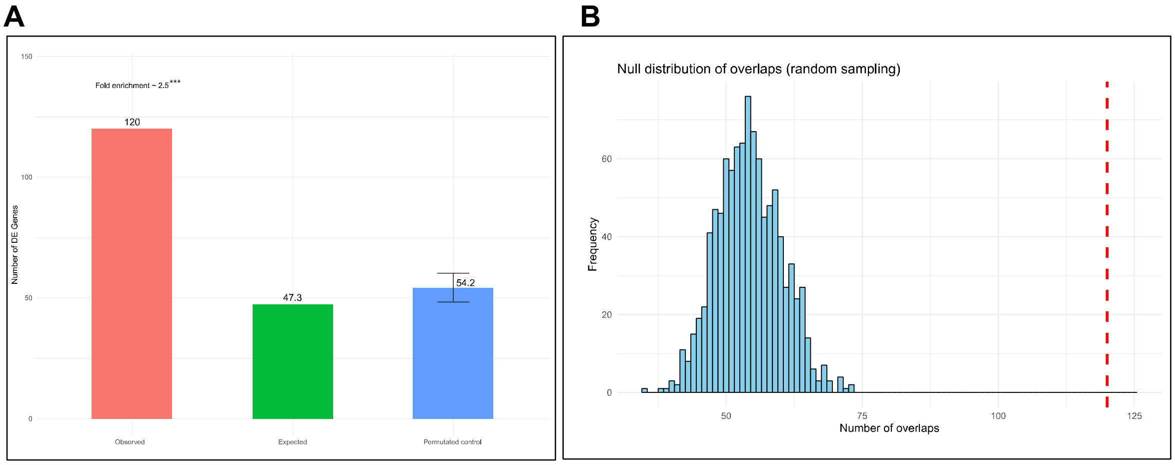

Supplemental Figure S8. Enrichment analysis of svRNA-TFO gene targets among differentially expressed genes (DEGs) relevant to SARS-CoV-2 infection in Calu-3 cells (GSE148729, GSE147507) and human lung tissues (GSE147507), and upregulated during severe COVID-19 (GSE171110). A) 120 DEGs were “Observed” to overlap with the 350 svRNA-TFO gene targets identified in this study (Supplemental Figure S6A-F). This represents a significant (***) ~ 2.5-fold enrichment (*p*-value < .001; Fisher’s exact test) when compared to the “Expected” number of DEGs based on a background rate of 13.5 %. See Panel B for the distribution of the “Permutated control”. **B)** Using the null gene target set, which corresponds to targets of 12 non-svRNA-TFOs detected in this study (see Materials and Methods), 1,000 groups of 350 gene targets were randomly selected to generate the “Permutated control”, for which the distribution is shown. This distribution was compared against the 120 DEGs that were “Observed” (represented as the vertical dashed red line), which represent *p*-value < .001 based on a one-sided test *t*-test. See also Supplemental Table S5.

Supplemental Table S5. Differentially expressed genes (DEGs) and svRNA-TFO target gene lists and annotations used in the enrichment analysis (see Supplemental Figure S7).

20260329_Supplemental_Table_S5.xlsx

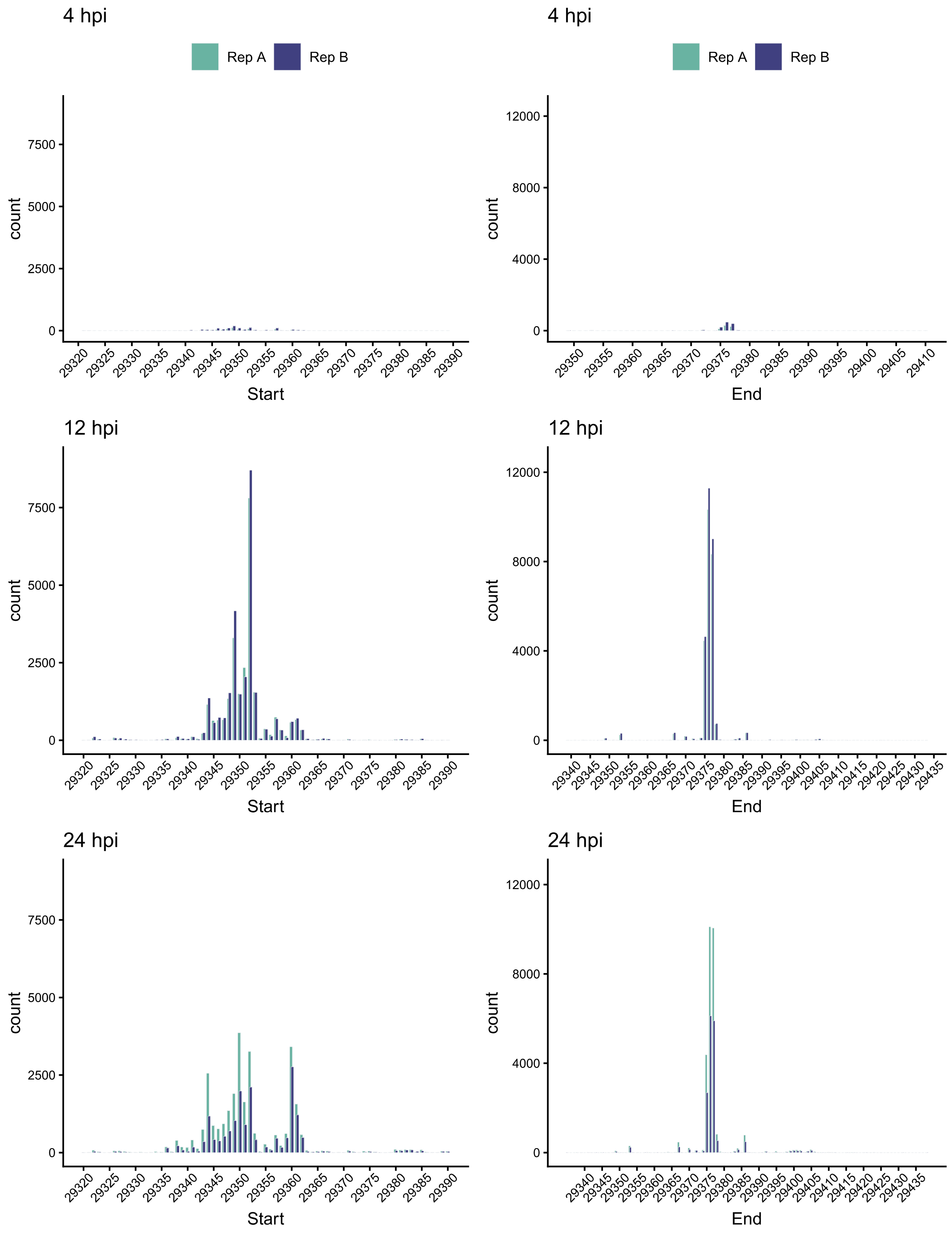

Supplemental Figure S9. Frequency of small RNA-sequencing read counts in proximity to the start and end of the svRNA-TFO-N region of interest. This re-analysis relied on small RNA-seq collected from Calu-3 cells at 4, 12, and 24 hpi with SARS-CoV-2 [1].

Supplemental Table S6. Base-centred counts of above-average short-BLASTN hits to the human genome (GRCh38) in identified regions of interest (nsp2, nsp3, S-ORF, E-ORF, N-ORF and 3' UTR) of SARS-CoV-2 (Wuhan-Hu-1) and SARS-CoV-1 (Tor2), using a multiple-test corrected significance threshold of *P*-value ≤ .05.

20260530_Supplemental_Table_S6.xlsx

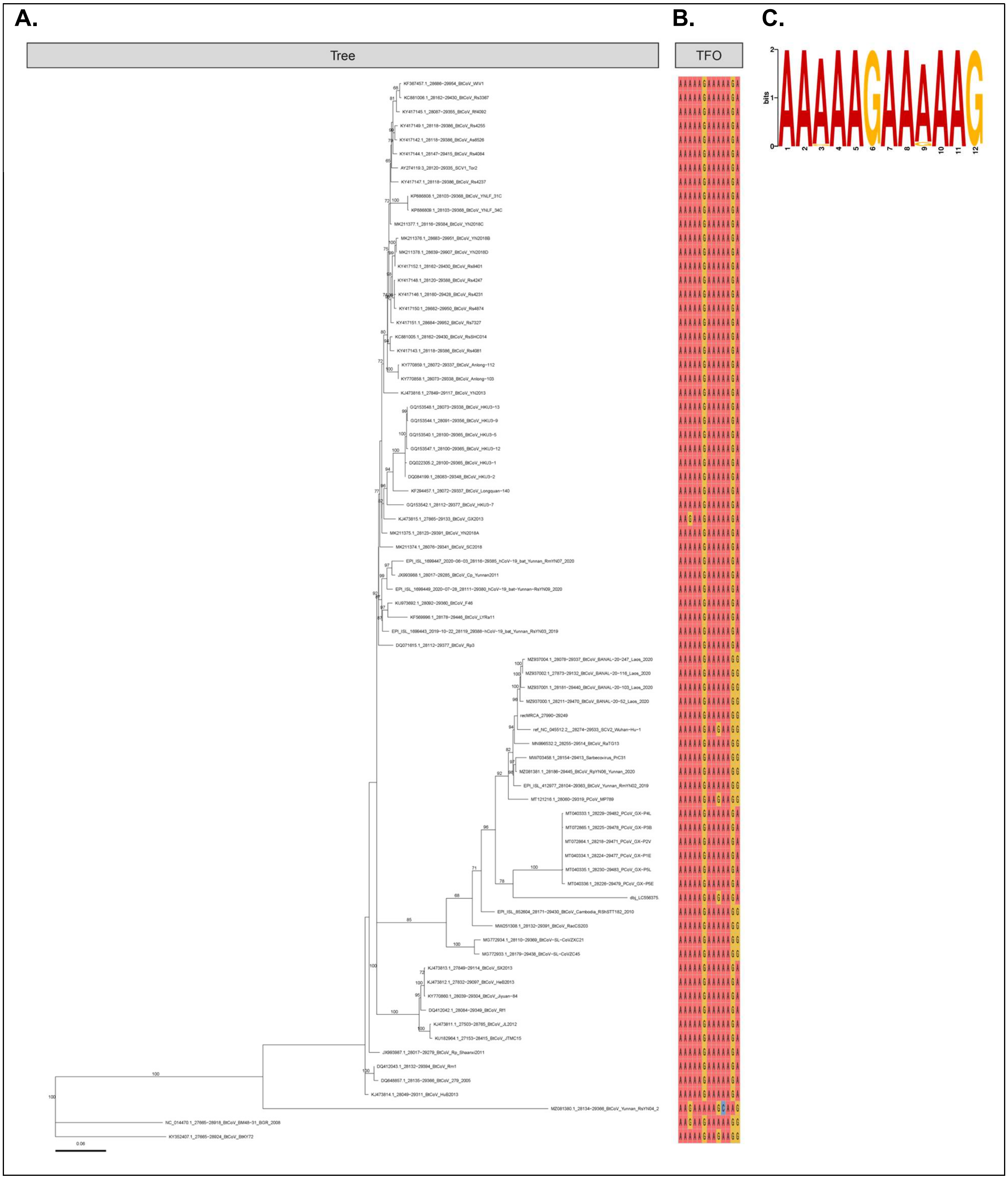

Supplemental Figure S10. Evolution of Nucleocapsid (N) open reading frame in *Sarbecovirus*. A) Phylogenetic reconstruction of aligned translated amino acids, with internal node confidence reported for values greater than 65 % out of 10,000 ultrafast bootstraps and re-rooted using Btk72 for visualization purposes. **B)** Sequence alignment segments showing polypurine triplex-forming oligonucleotide (TFO) region. **C)** Motif from 75 TFO-like sequences among *Sarbecovirus*.

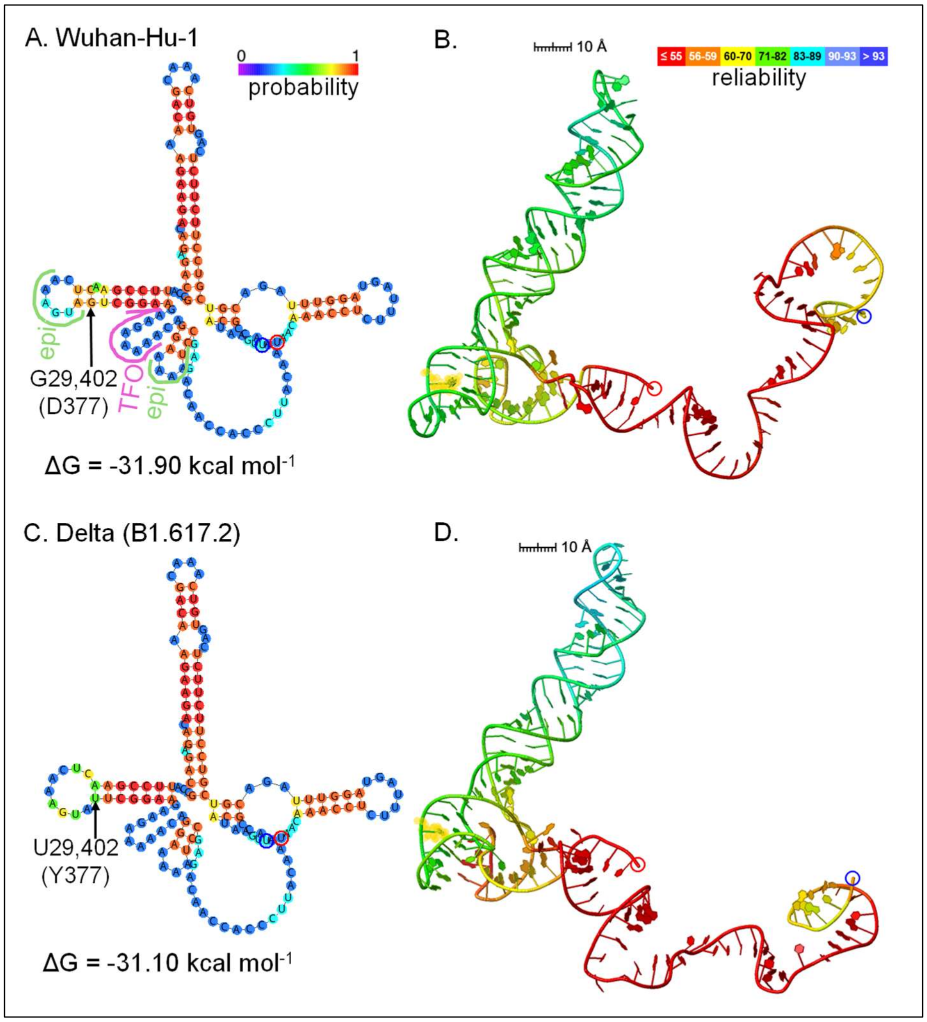

Supplemental Figure S11. Maximum Free Energy (MFE) of optimal and 3D models for SARS-CoV-2 svRNA-TFO-N precursor structure (29,343 – 29,492 in Wuhan-Hu-1). A) MFE of optimal structure for G29402 (D377) variant with base probability indicated by colour using Gibbs free energy (ΔG) and Turner energy model [4] at 37 °C. The AAGAA-like motifs identified in Kim et al. [5] are labelled green as 'epi' and triplex-forming oligo-nucleotides is labelled pink as "TFO". **B)** 3D model for the G29402 (D377) variant with base reliability indicated by colour (see methods). **C)** Same as A, but for U29402 (Y377) variant. **D)** Same as B, but for U29402 (Y377) variant. The 5′ and 3′ ends are highlighted in blue and red, respectively. Yellow highlighting in B and D denotes the G29402U mutation on the 3D models.

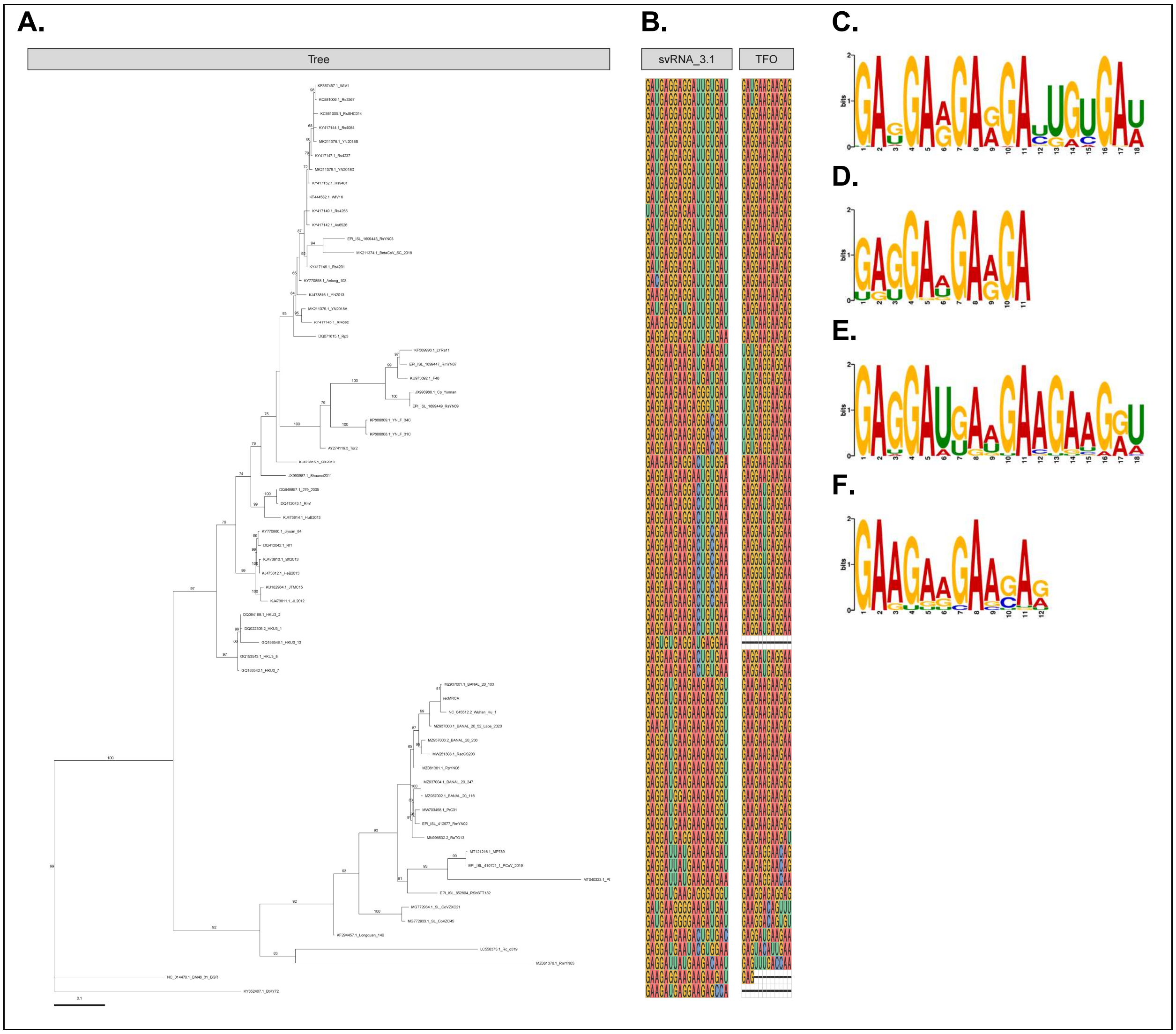

Supplemental Figure S12. Evolution of the nsp3 open reading frame (recombination region 2 only) in *Sarbecovirus*. A) Phylogenetic reconstruction of aligned translated amino acids, with internal node confidence reported for values greater than 65 % out of 10,000 ultrafast bootstraps and re-rooted using Btk72 for visualization purposes. **B)** Sequence alignment segments showing svRNA nsp3.1 from SARS-CoV-1 [6] and polypurine triplex-forming oligonucleotide (TFO) regions. **C to F**: Motifs for svRNA nsp3.1 and TFO regions from the SARS-CoV-1- (**C, D**), and SARS-CoV-2-related (**E, F**) sub-groups, respectively.

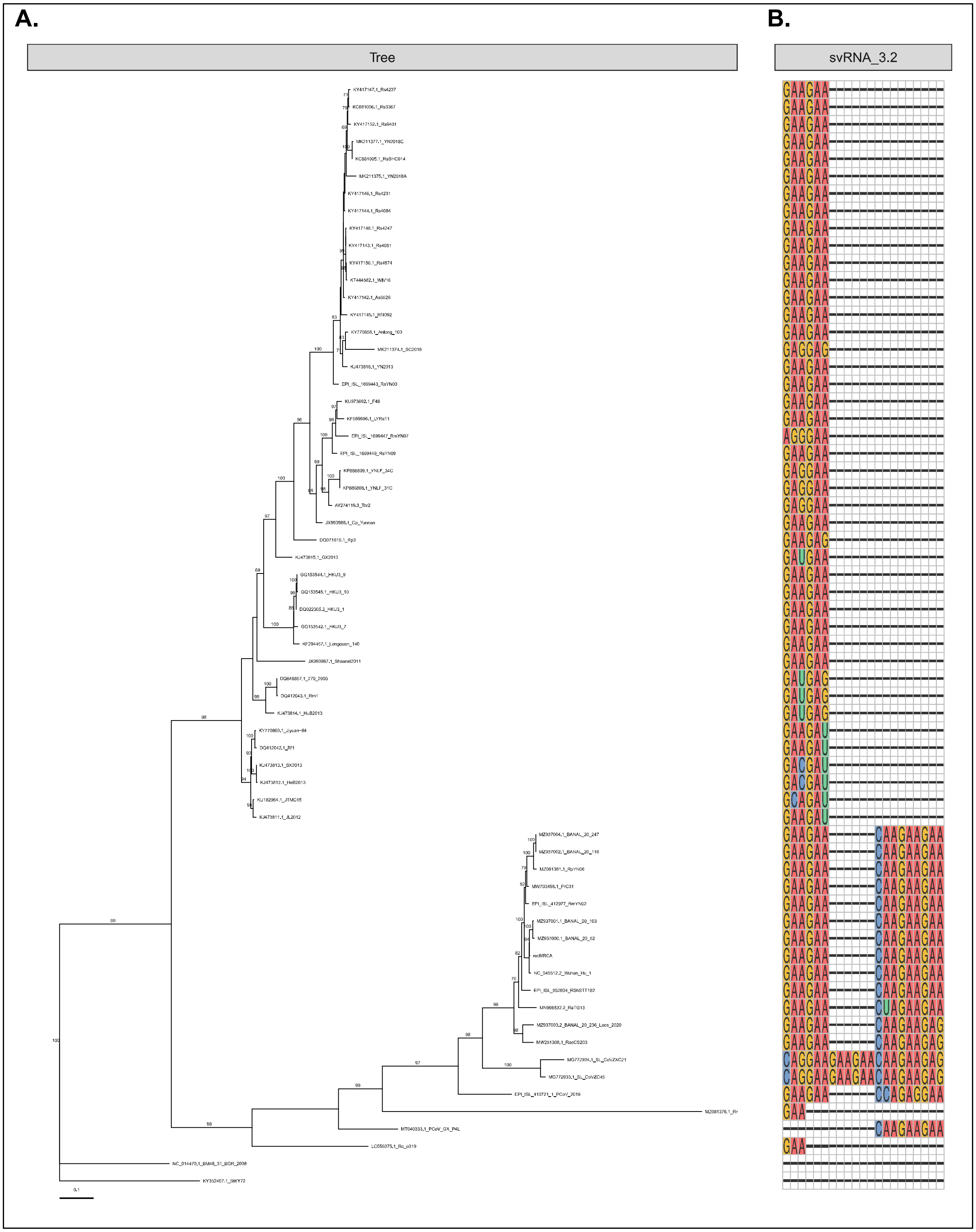

Supplemental Figure S13. Evolution of the nsp3 open reading frame (recombination region 3 only) in *Sarbecovirus*. A) Phylogenetic reconstruction of aligned translated amino acids, with internal node confidence reported for values greater than 65 % out of 10,000 ultrafast bootstraps and re-rooted using Btk72 for visualization purposes. **B)** Sequence alignment segments showing presence of an insertion among SARS-CoV-2-related viruses in the svRNA-nsp3.2 region from SARS-CoV-1 [6].

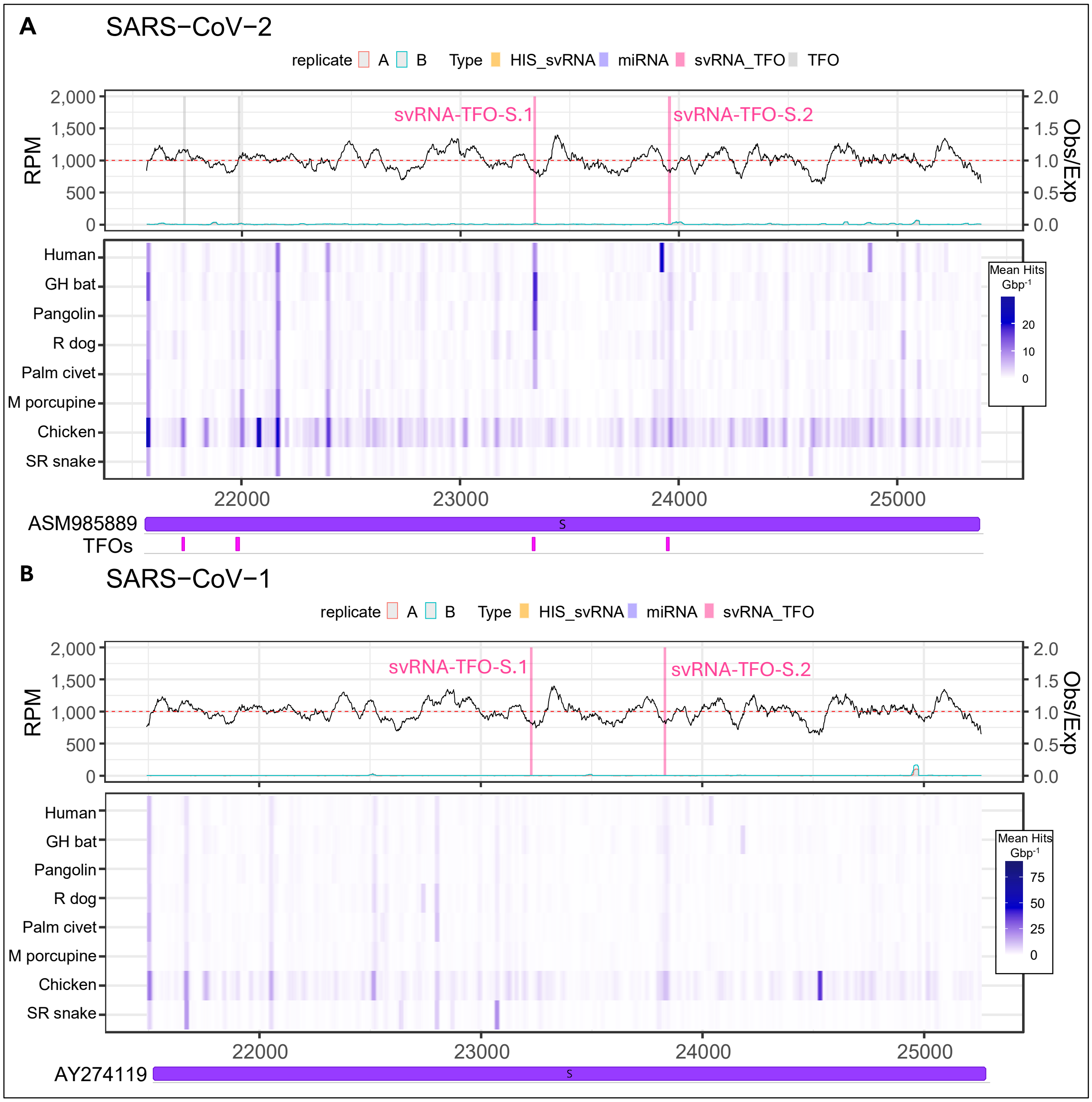

Supplemental Figure S14. Spike (S) open reading frame region integrating small RNA-sequencing (RNA-seq), synonymous-site conservation (SSC) analysis, triplex-forming oligonucleotide (TFO) predictions, and host genome homology. Topmost plots, first *y*-axis: reads per million (RPM) coverage small RNA-seq from Calu-3 cells 12 h post-infection [1]. Second *y*-axis: SSC analysis for *Sarbecovirus*, red dashed-line representing an equal ratio of observed (Obs) synonymous mutations compared to the number of expected (Exp). Middle heatmaps: rolling average BLAST hits against various animal genomes (GH bat = greater horseshoe bat; R dog = raccoon dog; M porcupine = Malaysian porcupine; and SR snake = San Diego ring-necked snake) (see Materials and Methods). Bottom feature plots: TFOs predicted against human lung enhancer sequences and svRNA-TFOs of concern identified using a functional genomics pipeline in this study (Supplemental Table S3). **A)** SARS-CoV-2 genetic region 21,563 – 25,384 in Wuhan-Hu-1 reference genome **B)** SARS-CoV-1 genetic region 21,492 – 25,259 in Tor2 reference genome.

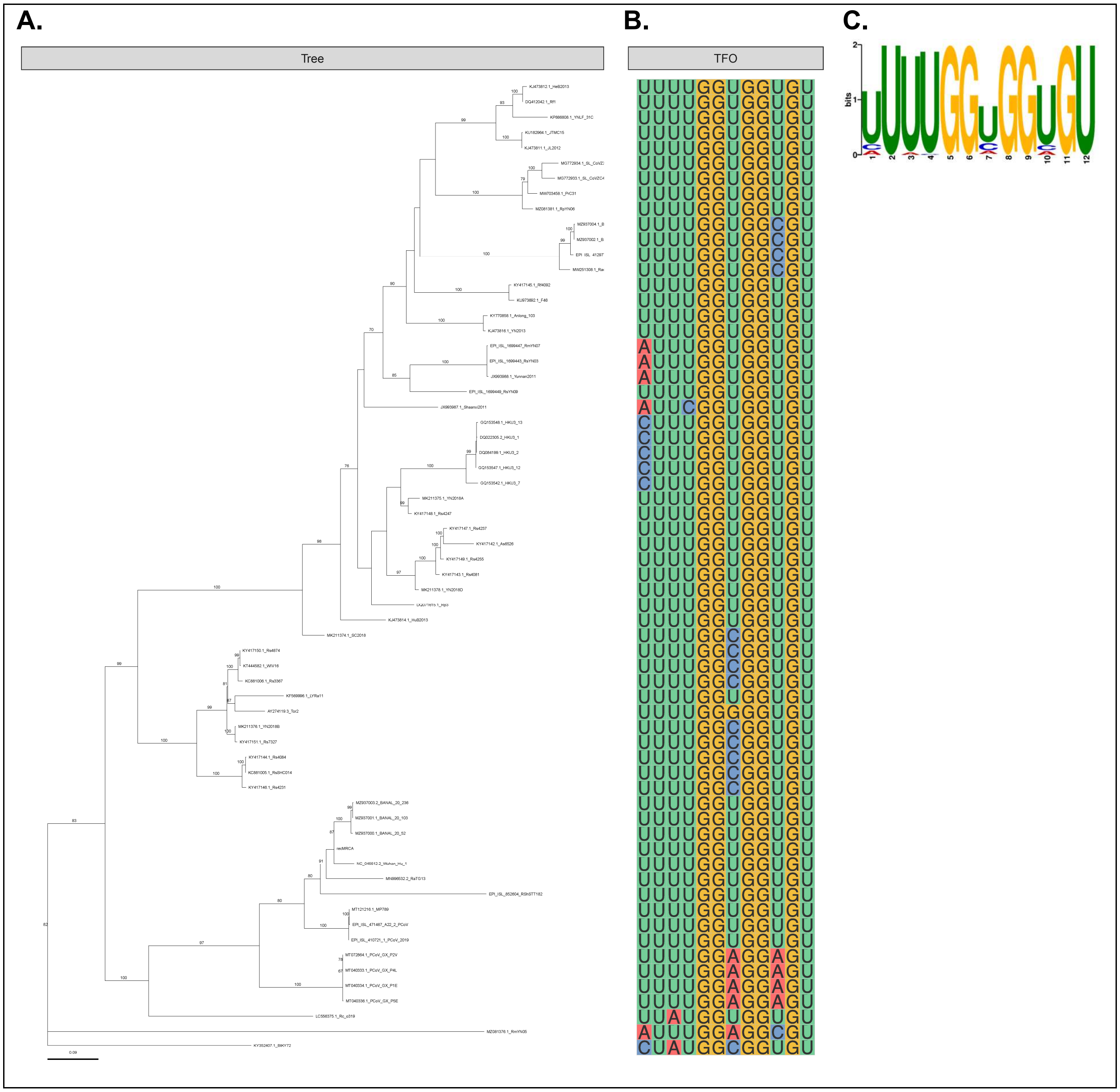

Supplemental Figure S15. Evolution of Spike (S) open reading frame (recombination region 16 only) in *Sarbecovirus*. A) Phylogenetic reconstruction of alignment translated amino acids, with internal node confidence reported for values greater than 65 % out of 10,000 ultrafast bootstraps and re-rooted using Btk72 for visualization purposes. **B)** Sequence alignment segments showing purine-pyrimidine triplex-forming oligonucleotide (TFO) associated with svRNA-TFO-S.1 in SARS-CoV-2 (see supplemental Figure S14). **C)** Motif from 64 TFO-like among *Sarbecovirus*.

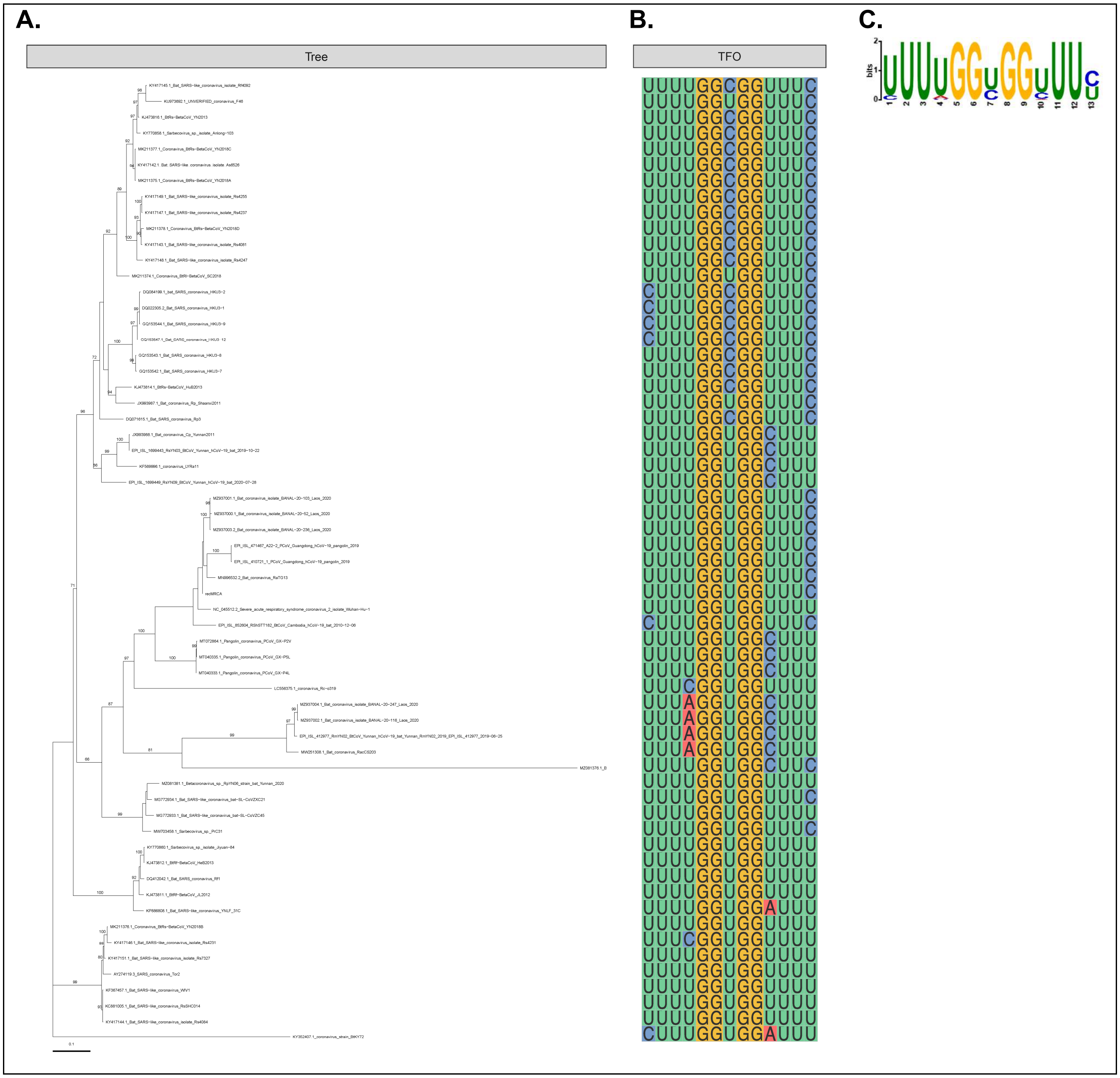

Supplemental Figure S16. Evolution of Spike (S) open reading frame (recombination region 17 only) in *Sarbecovirus.* A) Phylogenetic reconstruction of alignment translated amino acids, with internal node confidence reported for values greater than 65 % out of 10,000 ultrafast bootstraps and re-rooted using Btk72 for visualization purposes. **B)** Sequence alignment segments showing purine-pyrimidine triplex-forming oligonucleotide (TFO) associated with svRNA-TFO-S.2 in SARS-CoV-2 (see supplemental Figure S14). **C)** Motif from 61 TFO-like sequence among *Sarbecovirus*.

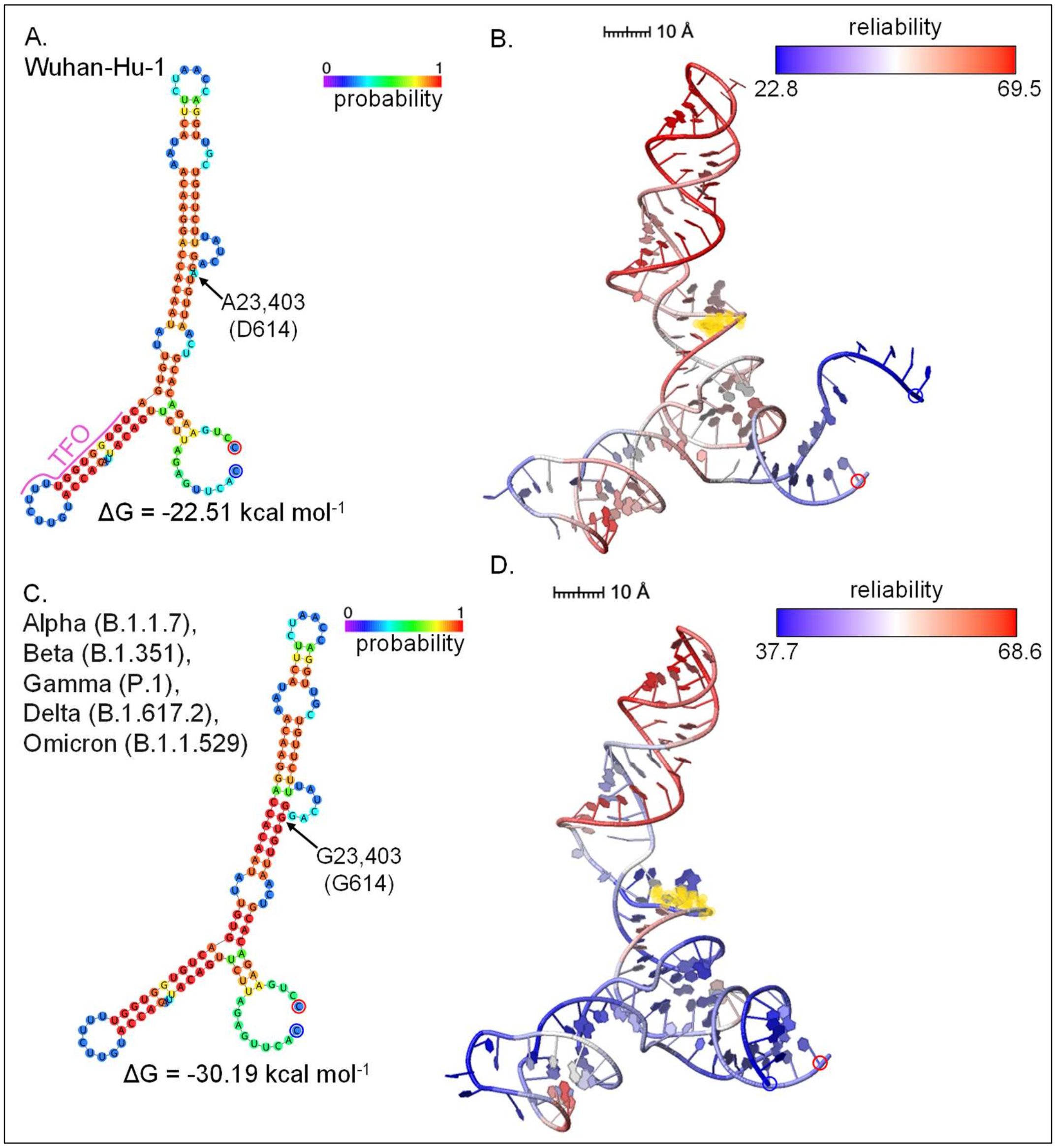

Supplemental Figure S17. Maximum Free Energy (MFE) of optimal and 3D models for SARS-CoV-2 svRNA-TFO-S.1 precursor structure (23,304 – 23,423 in Wuhan-Hu-1). A) MFE of optimal structure for A23403 (D614) variant with base probability indicated by colour using Gibbs free energy (ΔG) and Andronescu energy model [7] at 37 °C. The triplex-forming oligo-nucleotides is labelled pink as "TFO". **B)** 3D model for the A23403 (D614) variant with base reliability indicated by colour. **C)** Same as A, but for G23403 (G614) variant. **D)** Same as

B, but for G23403 (G614) variant. 5′ and 3′ ends are highlighted with blue and red circles, respectively. Yellow highlighting in B and D denotes A23403G (D614G) mutation on 3D models.

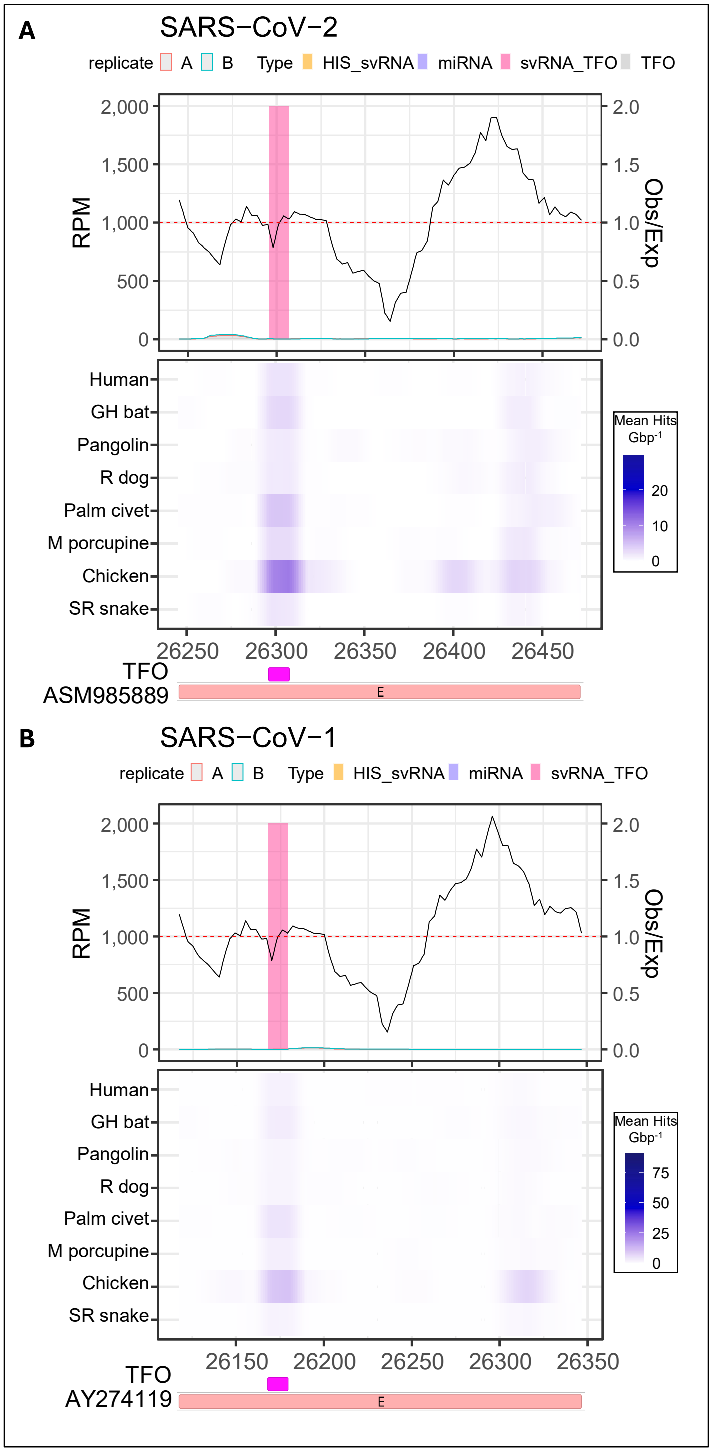

Supplemental Figure S18. Envelope (E) open reading frame region integrating small RNA-sequencing (RNA-seq), synonymous-site conservation (SSC) analysis, triplex-forming oligonucleotide (TFO) prediction, and host genome homology. Topmost plots, first *y*-axis: reads per million (RPM) coverage small RNA-seq from Calu-3 cells 12 h post-infection [1].

Second *y*-axis: SSC analysis for *Sarbecovirus*, red dashed-line representing an equal ratio of observed (Obs) synonymous mutations compared to the number of expected (Exp). Middle heatmaps: rolling average BLAST hits against various animal genomes (GH bat = greater horseshoe bat; R dog = raccoon dog; M porcupine = Malaysian porcupine; and SR snake = San Diego ring-necked snake) (see Materials and Methods). Bottom feature plots: TFO predicted against human lung enhancer sequence and svRNA-TFOs of concern identified using a functional pipeline in this study (supplemental Table S3). **A)** SARS-CoV-2 genetic region 26,245 – 26,472 in the Wuhan-Hu-1 reference genome. **B)** SARS-CoV-1 genetic region 26,117 – 26,347 in the Tor2 reference genome.

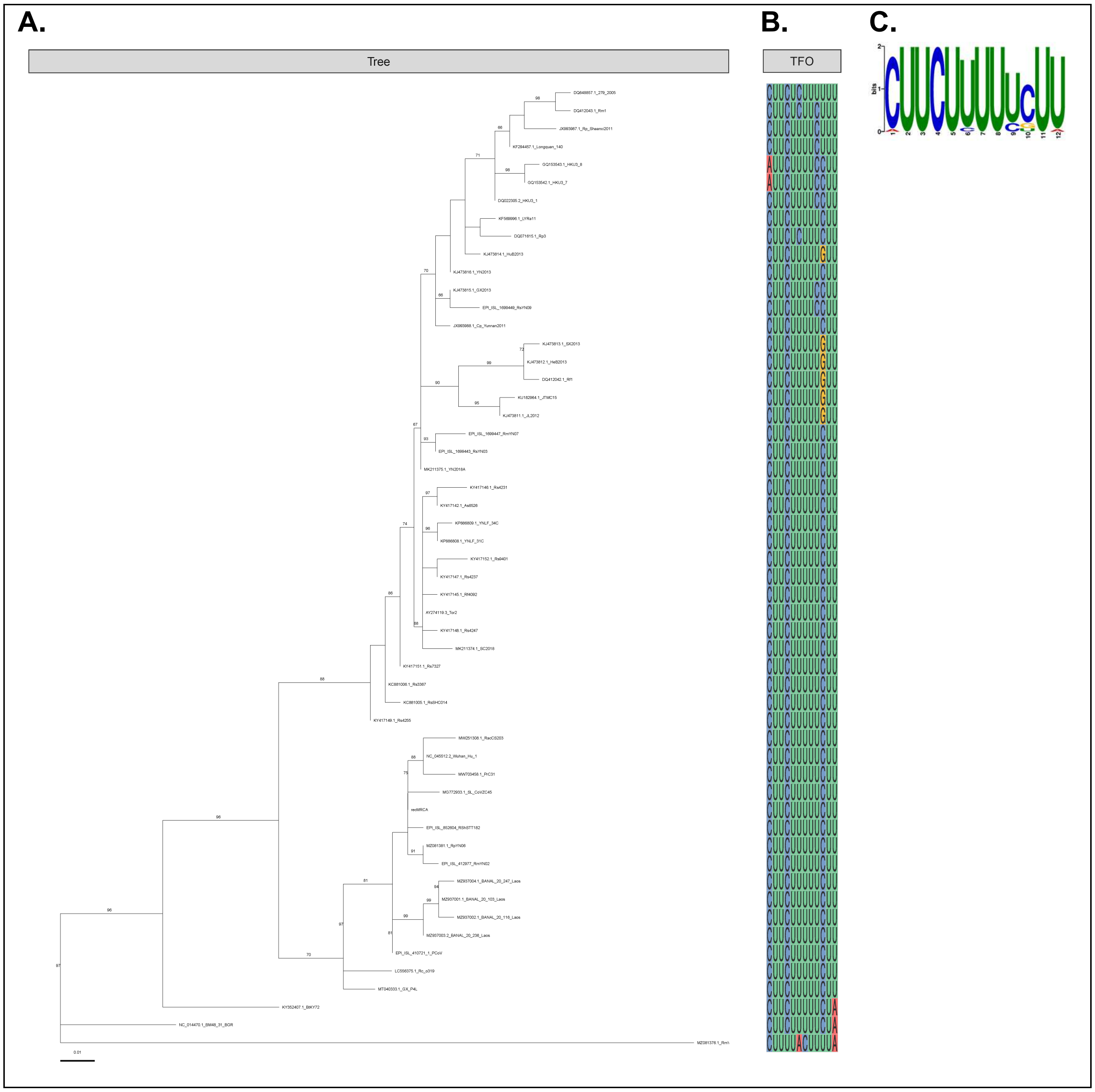

Supplemental Figure S19. Evolution of Envelope (E) open reading frame (includes recombination regions 19 and 20) in *Sarbecovirus*. A) Phylogenetic reconstruction of aligned translated amino acids, with internal node confidence reported for values greater than 65 % out of 10,000 ultrafast bootstraps and re-rooted using RmYN05 (MZ081376.1) for visualization purposes. **B)** Sequence alignment segment showing polypyrimidine triplex-forming oligonucleotide (TFO) associated with svRNA-TFO-E in SARS-CoV-2 (see supplemental Figure S18). **C)** Motif from 54 TFO-like sequences among *Sarbecovirus*.

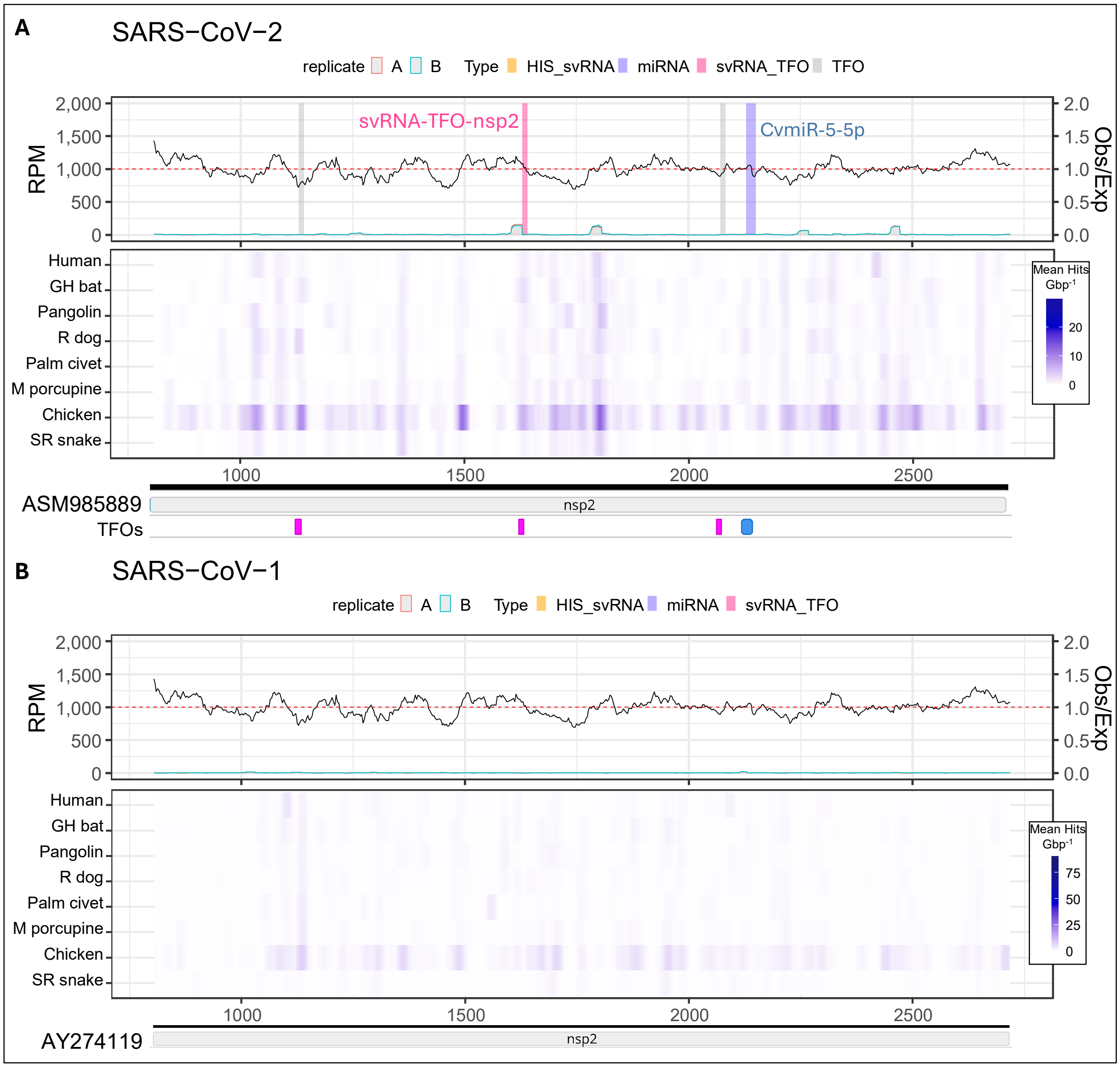

Supplemental Figure S20. nsp2 region integrating small RNA-sequencing (RNA-seq), synonymous-site conservation (SSC) analysis, microRNA (miRNA) annotations, triplex-forming oligonucleotide (TFO) predictions, and host genome homology. Topmost plots, first *y*-axis: reads per million (RPM) coverage small RNA-seq from Calu-3 cells 12 h post-infection [1]. Second *y*-axis: SSC analysis for *Sarbecovirus*, red dashed-line representing an equal ratio of observed (Obs) synonymous mutations compared to the number of expected (Exp). Middle heatmaps: rolling average BLAST hits against various animal genomes (GH bat = greater horseshoe bat; R dog = raccoon dog; M porcupine = Malaysian porcupine; and SR snake = San Diego ring-necked snake) (see Materials and Methods). Bottom feature plots: TFOs predicted against human lung enhancer sequences and svRNA-TFOs of concern identified using a functional genomics pipeline in this study (supplemental Table S3); and CvmiR-5-5p was identified by Zhao et al. [8]. **A)** SARS-CoV-2 genetic region 806 – 2,719 in Wuhan Hu-1 reference genome **B)** SARS-CoV-1 genetic region 805 – 2,718 in Tor2 reference genome.

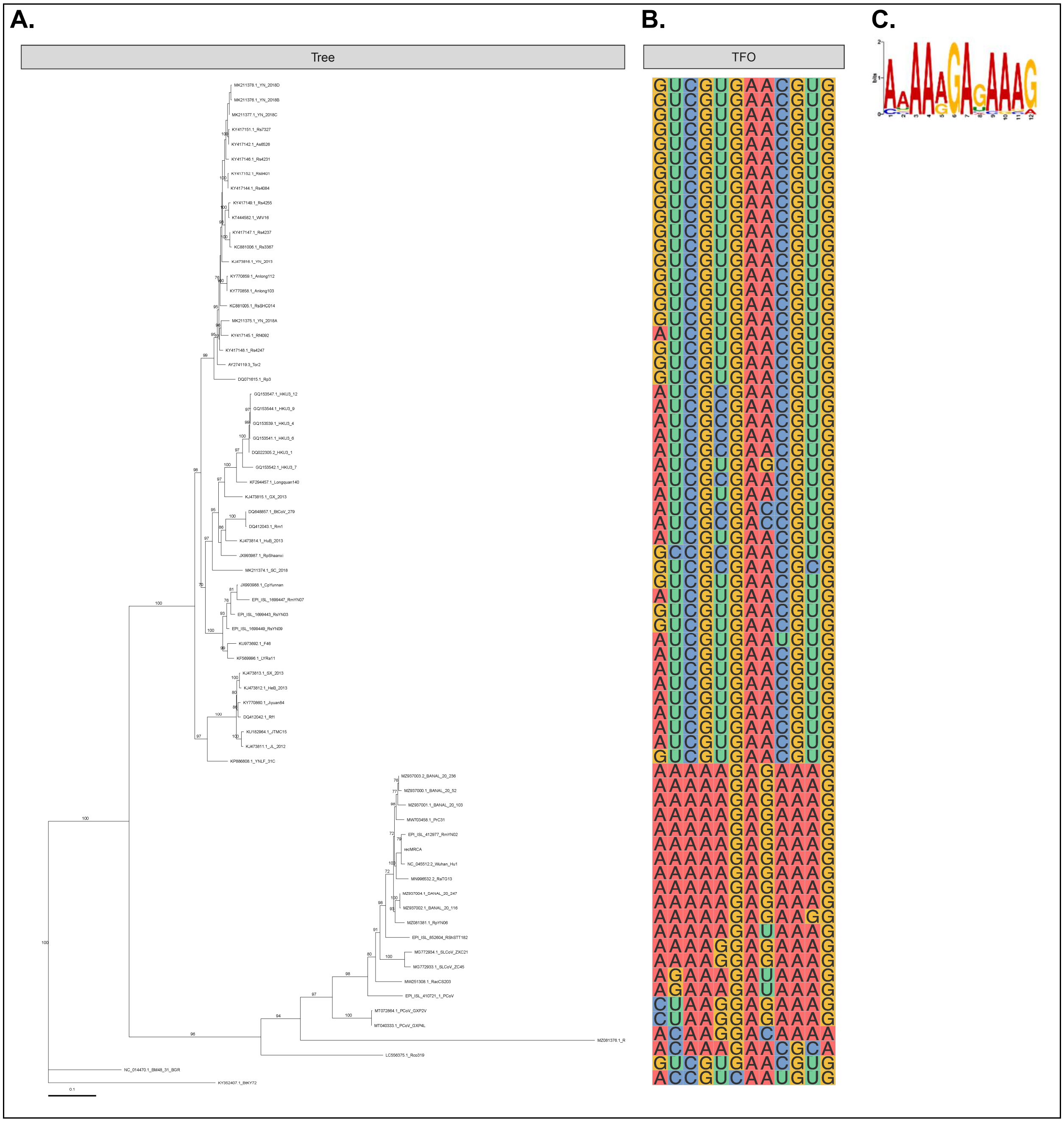

Supplemental Figure S21. Evolution of partial nsp2 region in *Sarbecovirus*. A) Phylogenetic reconstruction of aligned translated amino acids, with internal node confidence reported for values greater than 65 % out of 10,000 ultrafast bootstraps and re-rooted using Btk72 for visualization purposes. **B)** Sequence alignment segment showing polypurine triplex-forming oligonucleotide (TFO) associated with svRNA-TFO-nsp2 in SARS-CoV-2 (see supplemental Figure S20). **C)** Motif from 20 aligned TFO-like sequences among the SARS-CoV-2 group.

REFERENCES:

1. Wyler E, Mösbauer K, Franke V, Diag A, Gottula LT, Arsiè R, et al. Transcriptomic profiling of SARS-CoV-2 infected human cell lines identifies HSP90 as target for COVID-19 therapy. iScience. 2021;24: 1–18. doi:10.1016/j.isci.2021.102151
2. Blanco-Melo D, Nilsson-Payant BE, Liu WC, Uhl S, Hoagland D, Møller R, et al. Imbalanced host response to SARS-CoV-2 drives development of COVID-19. Cell. 2020;181: 1036-1045.e9. doi:10.1016/j.cell.2020.04.026
3. Lévy Y, Wiedemann A, Hejblum BP, Durand M, Lefebvre C, Surénaud M, et al. CD177, a specific marker of neutrophil activation, is associated with coronavirus disease 2019 severity and death. iScience. 2021;24. doi:10.1016/j.isci.2021.102711
4. Mathews DH. Using an RNA secondary structure partition function to determine confidence in base pairs predicted by free energy minimization. RNA. 2004;10: 1178–1190. doi:10.1261/rna.7650904
5. Kim D, Lee JY, Yang JS, Kim JW, Kim VN, Chang H. The architecture of SARS-CoV-2 transcriptome. Cell. 2020;181: 914-921.e10. doi:10.1016/j.cell.2020.04.011
6. Morales L, Oliveros JC, Fernandez-Delgado R, TenOever BR, Enjuanes L, Sola I. SARS-CoV-encoded small RNAs contribute to infection-associated lung pathology. Cell Host Microbe. 2017;21: 344–355. doi:10.1016/j.chom.2017.01.015
7. Andronescu M, Condon A, Hoos HH, Mathews DH, Murphy KP. Efficient parameter estimation for RNA secondary structure prediction. Bioinformatics. 2007;23: 19–28. doi:10.1093/bioinformatics/btm223
8. Zhao Q, Wang Q, Zhao B, Wang Y, Lü J, Guo Y, et al. Identification of a SARS-CoV-2 virus-encoded small non-coding RNA in association with the neurological disorders in COVID-19 patients. Signal Transduct Target Ther. 2022;7: 1–3. doi:10.1038/s41392-022-00969-1
